# Effect of antidiabetic drugs in Alzheimer´s disease: a systematic review of preclinical and clinical studies

**DOI:** 10.1186/s13024-025-00894-1

**Published:** 2025-10-27

**Authors:** Miriam Corraliza-Gomez, Maria Vargas-Soria, Monica Garcia-Alloza

**Affiliations:** 1 Division of Physiology. School of Medicine, Universidad de Cadiz, Edificio Andres Segovia C/Dr. Maranon 3, 3er piso, Cadiz, Spain; 2 Instituto de Investigacion E Innovacion en Ciencias Biomedicas de La Provincia de Cadiz (INIBICA), Cadiz, Spain

**Keywords:** Antidiabetic drugs, Alzheimer’s disease, Diabetes mellitus, Brain, Clinical studies, Preclinical studies

## Abstract

The potential neuroprotective effects of antidiabetic treatments have been largely assessed in Alzheimer’s disease (AD) and AD-like dementia models, with or without metabolic disorders. In this Line, these effects Have also been addressed in wide population-based studies or in patients with mild cognitive impairment, AD, diabetes or combined pathologies. Most common treatments include glucagon-like peptide 1 receptor agonists; thiazolidinediones; biguanides; sulphonylureas; dipeptidyl peptidase-4 inhibitors, insulin, amylin and others. To assess their impact, we have conducted a systematic search in PubMed to Identify studies addressing the effect of Antidiabetic treatments on AD or AD-like dementia preclinical models And clinical studies, yielding 3560 research items. After screening titles And abstracts, 380 papers met eligibility criteria (original full-text articles, written in English, focused on AD or AD-like dementia, involving antidiabetic treatments, containing data on neuropathological AD markers and/or cognitive function, and conducted in vivo or ex vivo), And 25 additional papers were added through citations, resulting in a Total of 405 primary research articles published between 1996 And 2024. We have reviewed the effects of antidiabetic treatments on tau pathology, neuronal health, oxidative stress and neuroinflammation, vascular alterations, implicated signaling pathways and cognitive function in AD and AD-like dementia preclinical models and patients. Overall, antidiabetic medications represent a promising therapeutic strategy to tackle neurodegeneration and cognitive decline in AD preclinical models. Nevertheless, further research is needed to optimize their clinical effectiveness.

**Graphical Abstract:**

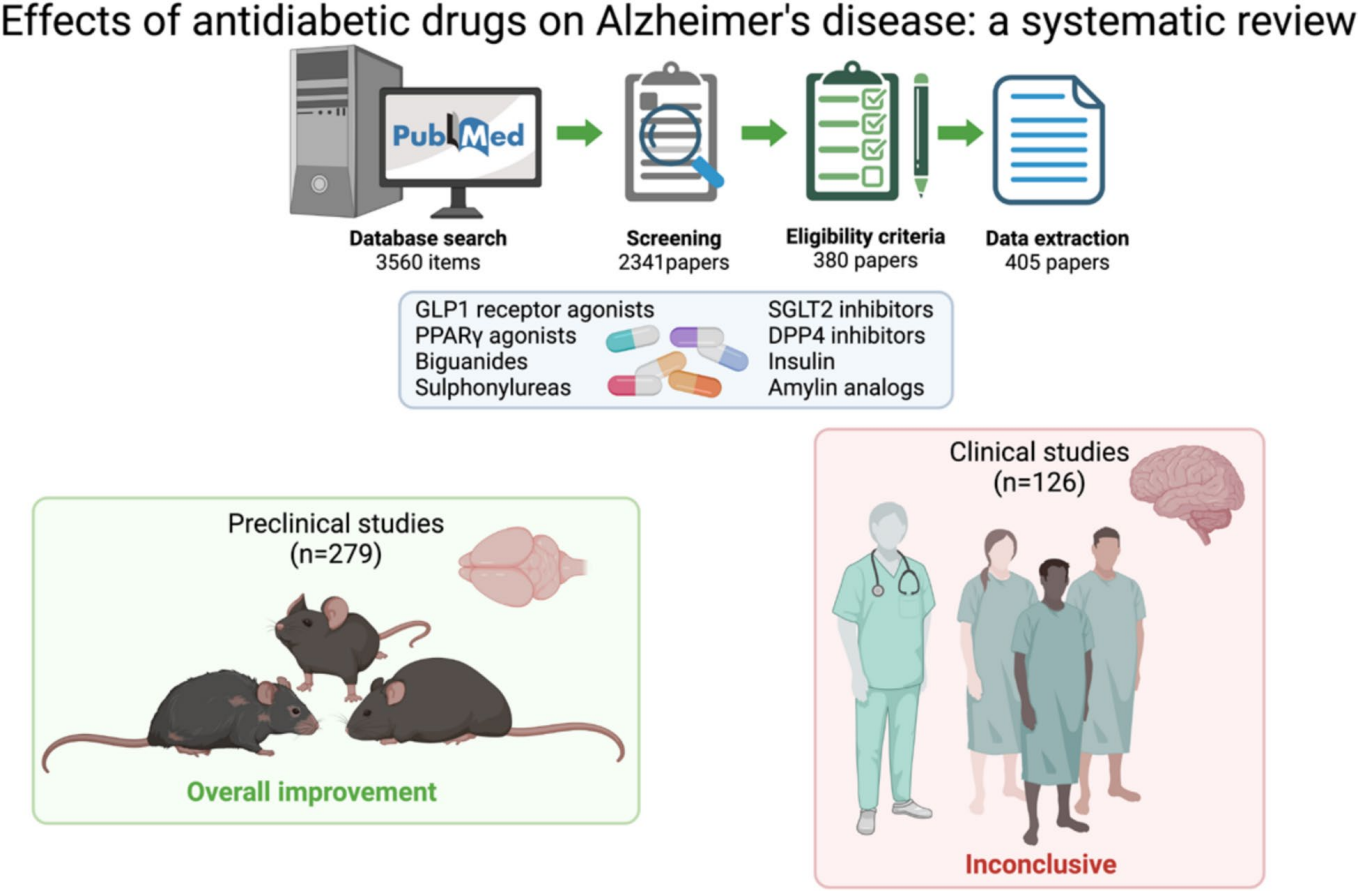

**Supplementary Information:**

The online version contains supplementary material available at 10.1186/s13024-025-00894-1.

## Background

Emerging evidence highlights significant overlap in pathological mechanisms of Alzheimer’s disease (AD) and type 2 diabetes mellitus (T2D) [1]. Both conditions exhibit disruptions in brain insulin signaling, crucial for neuronal functions such as glucose metabolism, synaptic plasticity, and the regulation of amyloid beta (Aβ) and tau proteins [2]. Chronic low-grade inflammation in T2D further exacerbates brain insulin resistance, promoting neuroinflammation, Aβ accumulation, tau hyperphosphorylation, and neuronal damage [1]. These connections, among others, suggest that antidiabetic medications may offer novel therapeutic strategies for AD [3]. Various antidiabetic treatments have been assessed in the context of mild cognitive impairment (MCI) and Alzheimer´s disease (AD), both alone and with metabolic disorders [4, 5]. These include: i) glucagon-like peptide-1 (GLP1) receptor agonists, like exenatide or liraglutide, which enter the brain rapidly and bind to neuronal GLP1 receptors; ii) thiazolidinediones (TZD), including pioglitazone and rosiglitazone, which enhance insulin sensitivity and act as agonists of the nuclear receptor peroxisome proliferator activated gamma (PPAR-γ); iii) biguanides, oral antidiabetics that ameliorate insulin sensitivity in insulin-resistant cases, like metformin; iv) sulphonylureas (SU), that stimulate insulin secretion by closing ATP-sensitive K^+^-channels in pancreatic ß-cells; v) sodium-glucose cotransporter 2 inhibitors (SGLT2i), which reduce glucose reabsorption in the kidneys, reducing glycemia; vi) dipeptidyl peptidase IV inhibitors (DPP4i), which increase GLP1 levels by inhibiting its degradation, therefore lowering glycemia; vii) insulin and insulin analogs, which lower glycemia by activating insulin signaling; and viii) other less common hypoglycemic agents like alpha-glucosidase inhibitors and meglitinides, which reduce glucose absorption or stimulate insulin release, and amylin and analogs, which suppress glucagon release [6].

This review systematically evaluates preclinical and clinical evidence on antidiabetic therapies, focusing on their effects on amyloid and tau pathologies, neuronal health, oxidative stress, neuroinflammation, vascular alterations, brain glucose metabolism and cognitive function. These treatments are also being explored for their potential to reduce dementia risk [4]. In this sense, it has been estimated that about half of T2D patients will develop dementia [7], and cognitive impairment in these patients complicate their ability to self-monitor glucose levels [8]. Given the rising prevalence of T2D and dementia in aging populations, this poses a serious threat to healthcare systems and the overall society. By providing a comprehensive assessment of antidiabetic treatments for AD, this review fills a gap in existing literature, offering new insights into their therapeutic potential. While these medications show promise for treating neurodegeneration and cognitive decline in AD, further research is needed to optimize their clinical application.

## Methods

This systematic review aimed to examine the impact of antidiabetic drugs on AD and AD-like dementia in preclinical models of neurodegeneration alone or with metabolic impairment, as well as in humans.

This systematic review was conducted in accordance with the Preferred Reporting Items for Systematic Review and Meta-Analyses (PRISMA) guidelines [9]. The review protocol was registered in the Prospective Register of Systematic Reviews (PROSPERO) under the registration number CRD420250653627. In brief, we conducted a systematic Literature review covering studies published from database inception until December 31st, 2024, using the Population, Intervention, Comparison and Outcome (PICO) strategy to explore whether antidiabetic treatments improve brain function in AD or AD-like dementia. The research question was: do antidiabetic treatments (I) benefit brain function (O) in AD or AD-like dementia models/patients (P), compared to untreated ones (C)? We systematically searched PubMed database, using the query “((Alzheimer OR dementia) OR ((Alzheimer OR dementia) AND prediabetes) OR ((Alzheimer OR dementia) AND diabetes)) AND antidiabetics”. Inclusion and exclusion criteria were jointly established by all authors, and studies were included if they fulfilled the following eligibility criteria: (1) original full-text articles; (2) written in English; (3) focused on AD or AD-like dementia, (4) involving antidiabetic treatments, (5) reporting on neuropathology and/or cognitive function, (6) conducted in vivo or ex vivo. Additional articles were included from citations. Exclusion criteria included: (1) non-original research (including reviews, comments, editorials, perspectives); (2) non-English language; (3) inaccessible or retracted publications; (4) studies involving natural compounds or lacking antidiabetic treatment; (5) co-treatments with other drugs in preclinical studies; (6) studies not focused on AD-related dementia, or including comorbidities unrelated to metabolic impairment; and (7) in vitro studies.

For the study selection process, two authors were responsible for screening the titles and abstracts of all records and determining which articles to include. If needed, the third author was consulted to make the final determination regarding an article's inclusion. The selected studies were categorized into preclinical and clinical groups and classified with subheadings to structure the review. Data collection was performed independently by each researcher for each section. Supplementary Table 1 summarizes the report details (author, year, publication source), study characteristics (sample details) and research design (pharmacological treatment).

## Results

### Description of studies included

**Fig. 1:**
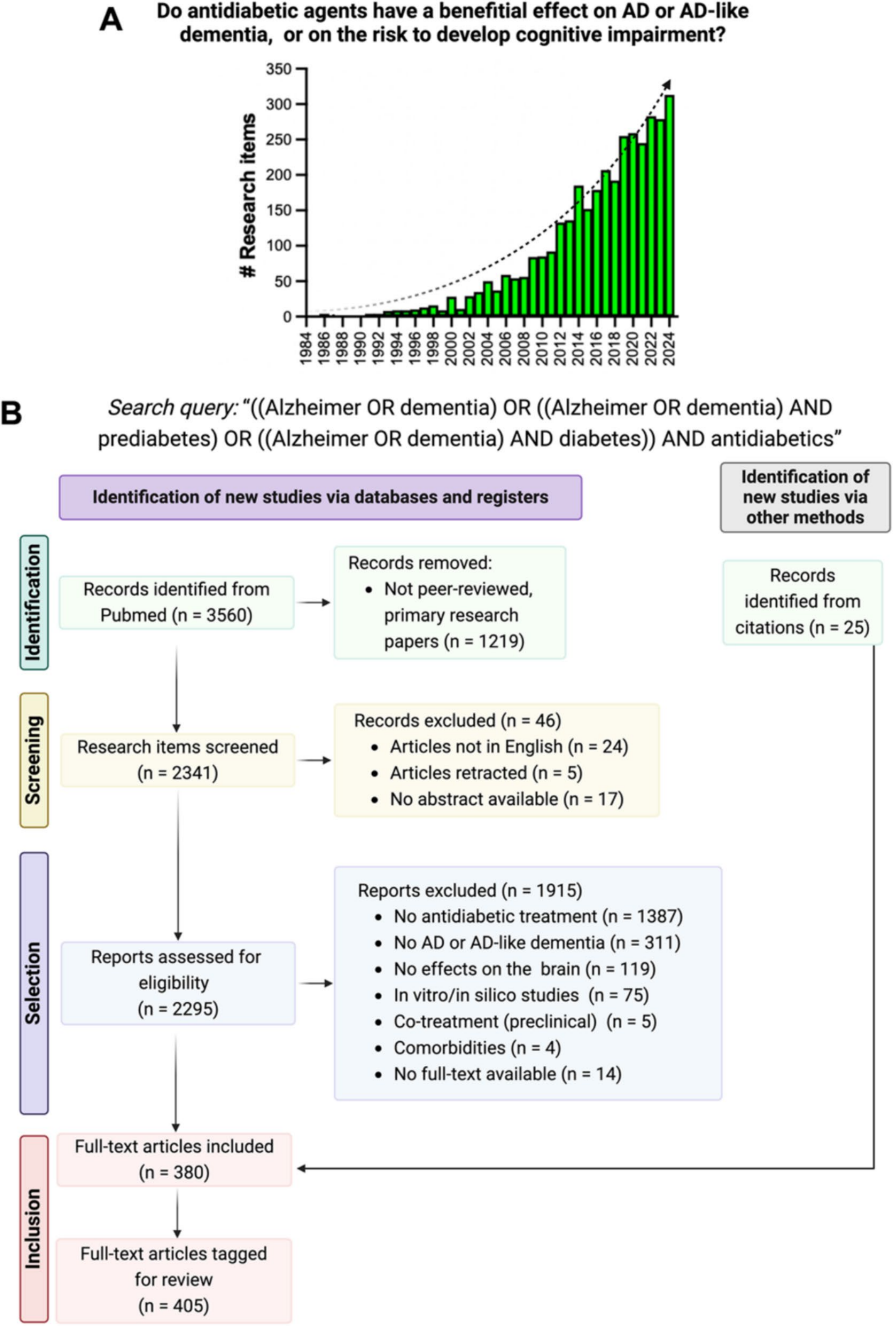
Literature search and study selection process: PRISMA flow diagram.** A** Timeline for the last 40 years of research items recovered by PubMed using the search query defined for our systematic review. **B** PRISMA 2020 flowchart of study selection and screening. Created with BioRender.com

Our search identified 3560 records published between 1945 and 2024, showing an exponential increase in the number of articles addressing our research question over time (Fig. 1A). After excluding non-primary research (*n* = 1219), 2341 articles remained. A first title/abstract screening excluded 46 records, leaving 2295 articles for further assessment. Of these, 380 met the eligibility criteria, and 25 additional papers were added through citation, resulting in 405 studies published between 1996 and 2024 (see Supplementary Table 1). Figure 1B illustrates the review workflow.

We summarized all current evidence on the effects of antidiabetic treatments on AD or AD-like dementia, categorizing studies into: i) preclinical models of classical AD pathology (amyloid and tau), ii) preclinical models of sporadic AD/AD-like dementia, iii) preclinical models of metabolic impairment, iv) preclinical models of AD concomitant with metabolic impairment and v) clinical studies involving AD/AD-like dementia patients.

### Preclinical models reproducing classical AD pathological features: amyloid and tau pathologies

Due to the methodological heterogeneity across preclinical studies, the experimental paradigms of different studies – including design, species and disease models – are summarized in Supplementary Table 2.

#### Murine models reproducing amyloid-ß (Aß) pathology

**Fig. 2:**
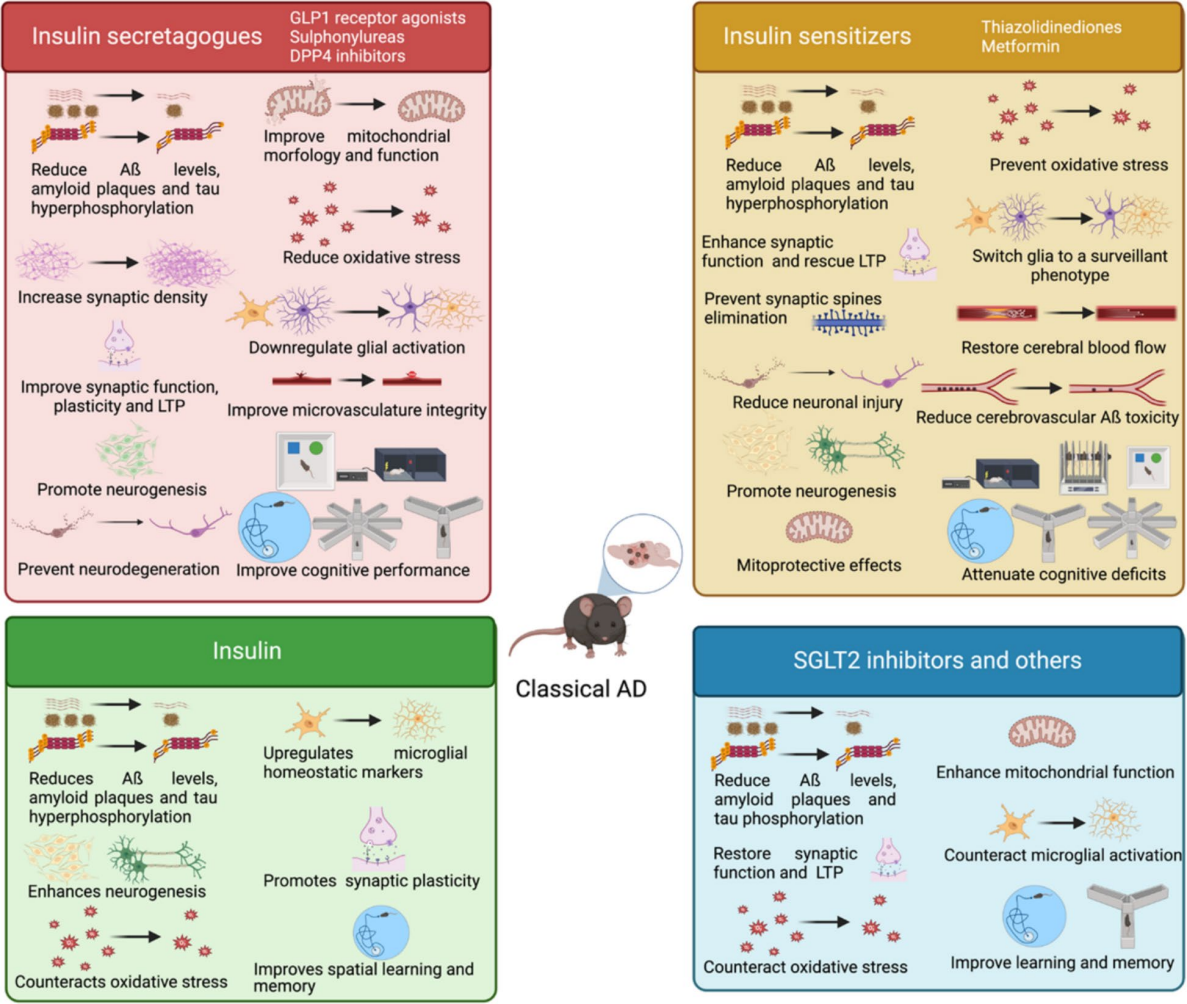
Protective effects of antidiabetic drugs as disease-modifying therapies for dementia on preclinical models of classical AD pathology. Created with BioRender.com

This section summarizes studies on transgenic mice overexpressing human amyloid precursor protein (APP) with various mutations, as well as Aß injected via intracerebroventricular or intrahippocampal routes. Major findings are summarized in Fig. 2and bibliographic references are compiled in Tables 1 and 2.

**Table 1:** Summary of preclinical studies assessing the effects of antidiabetic drug groups across different experimental models. Cell values indicate the number of studies as well as the specific references on a drug group within each experimental model

	**Classical AD pathology**	**Sporadic AD and AD-like dementia**	**Metabolic impairment**	**Cognitive and metabolic impairment**
**GLP1 receptor agonists**	44 [10–53]	11 [54–64]	13 [65–77]	4 [78–82]
**TZD/PPAR-γ agonists**	35 [83–117]	18 [118–135]	9 [136–144]	1 [145]
**Biguanides**	12 [146–157]	26 [158–183]	18 [73, 184–200]	2 [201, 202]
**SGLT2i**	-	5 [203–207]	4 [193, 208–210]	1 [211]
**DPP4i**	8 [212–219]	4 [164, 220–222]	6 [208, 223–227]	2 [228, 229]
**Sulphonylureas**	3 [101, 230, 231]	1 [232]	4 [185, 187, 232, 233]	-
**Insulin**	19 [234–252]	18 [253–270]	9 [66, 185, 271–277]	2 [278, 279]
**Amylin and analogs**	8 [280–287]	1 [171]	1 [80]	-
**Alpha-glucosidase inhibitors**	-	-	-	1 [288]

**Table 2:** Positive effects and molecular mechanisms of antidiabetic drugs in preclinical studies at the brain level

Group	Mechanism of action in diabetes	Brain delivery potential	Molecular mechanisms in AD and AD-related pathology
**GLP1 agonists**	Enhance insulin secretion and suppresses glucagon release	High [289]	**AD neuropathological hallmarks and markers** • ↓ Aß levels (Aβ40 And Aβ 42, soluble AB, amyloid plaques, oligomers) [10–13, 15–28, 30, 32, 42, 49–52, 79, 81, 82]. • ↓ amyloidogenic pathway: ↓ BACE1, PSEN1, APP expression and ↑ α-secretase [32] • ↓ tau and neurofilament-H and -M phosphorylation [24, 25, 33, 34, 50, 66–70, 79, 81, 82] • ↑ synaptic density (↑ PSD95, NeuN, synaptophysin, synapsin-1, MAP2, BDNF and glutamatergic receptors) [11, 19–22, 32, 33, 35, 36, 53, 62, 78] • Protect again neuronal apoptosis (↓ cleaved caspase 3) [62] • Improve synaptic structure (↓ synaptic cleft width and hippocampal ultrastructural damage) [11, 267] • ↑ LTP and ↓ pyramidal neuron over-excitability [19–22, 25–27, 38–42, 53, 73, 74] • ↑ cholinergic activity (↑ Ach and ChAT and ↓ AchE activity) [12] • Restore chemical synapses (↑ adrenaline, noradrenaline and dopamine levels) [33] • ↑ neurogenesis, neuroblast differentiation and neuronal density [16, 17, 19–22, 28, 43, 71, 72] Oxidative stress and neuroinflammation • Mitoprotection (enhance mitochondrial morphology, ↓ *Drp1* and *Fis1,* ↑ *Opa1* and *Mfn2)* [11, 36] • Improve mitochondrial bioenergetics (↑ ATP levels and complex I activity) [11, 12, 36]. • ↓ Oxidative stress (↓ MDA, ROS, 4-HNE, 8-OHdG, nitrite, TBARS and carbonyl groups; ↑ SOD) [28, 44, 51, 65, 73] • Modulate microglia phenotype (↓ Iba1, ↓*Cd86/cd206*, ↓ *Apoe*, *Ccl4*, *Ccl6*, *Cd9*, *Cd52* and *Timp2*) [16, 18–23, 27, 28, 30, 35, 49, 58, 63, 64, 79] • Modulate astrocytes phenotype (↓ GFAP, *Lcn2*, *Osmr* and *Ggta1*) [15, 16, 18, 23, 27, 28, 30, 32, 35, 58, 63, 73] • Modulate cytokines: ↓ pro-inflammatory TNF-α, IL-1ß, *Ifng, Il6, C1q;* ↑ *Il4* and IL-10. [27, 32, 49, 61] **Vascular damage and brain glucose metabolism** • ↑ LDH activity and β-hydroxybutyric acid and lactic acid levels [44] • Improve cerebral vascular and microvascular integrity, ↓ microaneurysms and cerebral extravasations [45, 63, 64, 76] • Improve BBB function (restore occluding expression, ↓ vascular leakage) [64, 76] • Restore glucose-6-phosphate dehydrogenase activity, ↓ cerebral pyruvate and lactate levels [51] • ↓ cortical hemorrhage [79] **Brain signaling pathways** • ↑ insulin levels [66, 67, 69] • Regulate insulin pathway genes (IR, IRS1/2, AKT1/3, IGF1R, Wnt/ß-catenin) [10, 13, 18, 29] • Activate the PI3K/AKT/GSK3ß pathway [12, 18, 25, 33, 35, 57, 58, 60, 65–70, 77] • ↓ GSK3ß activation [12, 18, 25, 33, 35, 58, 65, 67] • ↑ cAMP/PKA/CREB signaling (↑ cAMP levels and PKA and CREB phosphorylation [15, 36, 39, 40] • Anti-apoptotic effects (↓Bax And cleaved caspase 3, ↑ BCL2) [46, 77, 89] • Alleviate calcium overload (↑ calcium/calmodulin-dependent protein kinase II) [47] • ↑ GLP1 and GLP1 receptor levels [32, 33, 51, 56, 60, 68, 70, 72, 77] • ↑ AQP4 localization around blood vessels and ↑ water flux into the glymphatic system [15] • ↓ endoplasmic reticulum stress (regulating autophagy-related proteins, ↓p62) [35, 51, 77] • Modulate MAPK pathways (activating ERK1 and deactivating JNK1/2 and PKA) [50, 51, 59] • ↑ protein-tyrosine phosphatase 1B levels [56]
**TZDs and other PPAR-γ agonists**	Activate PPAR-γ, improve insulin sensitivity	Limited [290]	**AD neuropathological hallmarks and markers** • ↓ Aß levels (Aß40 and Aß42, soluble and non-fibrillar Aß, amyloid plaques and Aß oligomers) [83–86, 90, 92–99, 116, 118, 123, 124, 136, 138, 145] • ↓ amyloidogenic pathway: ↓ APP expression, ↑ α-secretase and ↓ BACE1 and presenilin-1) [84, 85, 91, 97, 136, 137] • Enhance Aß transport (↑ ABCA1 and LRP1 receptor expression, ↑ ApoE levels and its lipidation index) [86, 92, 100, 106, 229, 279] • Boost Aß phagocytosis by glial cells (↑CD36 in microglia, ↑CD11b colocalization with plaques) [86, 92, 99, 100] • ↑ Aß degradation (↑ IDE expression and activity) [94, 96, 138] • ↓ tau phosphorylation [92, 101, 116, 124, 145] • Enhance synaptic function and plasticity (↑ synaptophysin, vesicle-associate membrane proteins, PSD95) [95, 102, 124] • Prevent synaptic spines elimination and rescue dendritic spine [103, 121] • ↑ cholinergic activity (↑ Ach and ChAT, ↓ AchE activity) [87, 91, 102, 125–127, 137, 139] • Improve LTP and LTD, enhances short- and long-term plasticity and reverts excitability [104–107, 116, 120, 141] • ↓ neuronal loss and white matter thinning [97, 124] • Restore calcium homeostasis (↓ Ca^2+^-dependent afterhyperpolarization) [119] • ↑ neurotrophic factors *Creb*, *Bdnf*, *Gdnf* and *Ngf* [97, 140, 291, 292] **Oxidative stress and neuroinflammation** • Promote mitochondrial biogenesis (↑ estrogen-stimulated related receptor α, ↓PPAR-γ cofactor 1α) [122] • ↓ Oxidative stress (↑ SOD1 and peroxiredoxin-5; ↑ glutathione, SOD and catalase activities; ↓ ROS promoting proteins COX-2 and iNOS; ↓MDA, lipid peroxidation and nitrite levels) [84, 102, 108, 109, 125, 126, 129, 130, 137, 139, 142, 291, 292] • Improve mitochondrial bioenergetics (↑ complex I, complex II and complex IV enzyme activities) [102]. • Modulate microglia phenotype (↓ Iba1, ↑ Ym1, Fizz1 and Arg1) [83, 85, 86, 88, 92, 95, 96, 100, 110] • Modulate astrocytes phenotype (↓ GFAP) [85–88, 95] • ↓ pro-inflammatory cytokines (IL-1ß, IL-6, IL-12, TNF-α, IFN-γ, MCP1, MCSF and RANTES) and genes (*Tnfaip2*, *Tnfrsf1a*, *Il1rap*, *C1ql3*, *C1ql2* and *NFκB*) [86, 98, 100, 102, 111, 116, 124, 126, 291, 292] • ↓ NFκB activation, AGEs and RAGE levels [142] • ↑ anti-inflammatory cytokines (IL-10, IL-1α and IL-7) and genes reducing cytokine signaling (*Socs2*, *Socs5*, and *Lpin1*) [116, 124] **Vascular damage and brain glucose metabolism** • Normalize cerebrovascular reactivity, dilatory responses and proteins related to vascular tone (↓ SOD2 and ↑ NO) [45, 87, 88, 108] • Restore neurovascular and neurometabolic coupling and cerebral blood flow [84] • ↑ brain LDH activity [44] • ↓ pyruvate to lactate conversion and restore brain metabolites to wildtype levels [84, 122] • Improves brain glucose uptake and metabolism (↑ GLUT 1 and GLUT4) [98, 152] **Brain signaling pathways** • Restore insulin/IGF1/AKT/GSK3ß signaling [124, 131, 138, 141] • ↓ GSK3ß activation [124, 131]. • ↓ apoptosis (↓ p25, p35 and ↓ caspase-3 levels and activity) [105, 126] • ↑ Dvl3 and restore Wnt and ß-catenin levels [91, 95] • ↑ glucocorticoid receptors [112] • ↑ SNARE-associated proteins (VAMP2, CPLX2, PACS1, piccolo, vesicle-fusing ATPase) [106] • Stimulate (ERK)/MAPK pathway and PPAR-γ activity [106, 113] • Activate coactivator 1α/GLUT4 signaling [98]
**Biguanides (metformin)**	Increase insulin sensitivity, inhibit hepatic gluconeogenesis	High [293]	**AD neuropathological hallmarks and markers** • ↓ Aß levels (AB40 and AB42, insoluble AB42, amyloid plaques). [146–149, 154, 158, 161, 162, 168, 184, 185, 187, 190, 202] • ↓ amyloidogenic pathway (↓ BACE1 and APP) [146, 147] • ↑ IDE expression [149, 187, 202] • ↓ tau phosphorylation and tau aggregates spreading [148, 154–156, 158, 160, 162, 168, 170, 184–186, 188–190] • Promote neurogenesis (↑ Bdnf and Ngf, Dcx and Ki67), increasing neurotrophic factors CREB, BDNF, NGF and GDNF [147, 149, 164, 184, 191, 194] • ↑ neuronal density and preserve Nissl bodies [55, 147, 149, 151, 160, 161, 173, 174, 176–178, 188, 189] • ↑ cholinergic activity (partially restore Ach, ↓ AChE levels and activity). [151, 168, 177, 179, 181, 185, 187, 191] • ↑ dopamine levels [168, 193] • Mitigates excitotoxicity. (↓ NMDA receptor 2 A) [184] • Boost synaptic function and connectivity and LTP (↑ synaptophysin, PSD95, GluR1, NR2A and NR2B, synapsin-1) [162, 164, 165, 179, 190, 201] **Oxidative stress and neuroinflammation** • ↓ Oxidative stress: ↑ SOD, glutathione and antioxidant status, catalase and glutathione transferase activities; ↓ MDA, TBARS, lipid peroxidation, NADPH oxidase, COX2, iNOS, NO, nitrite, nitrate, AGEs, RAGE [149, 159, 168, 170, 177, 178, 181, 184–187, 195, 197] • Enhance mitochondrial ATP production and coupling [196] • Modulate microglia phenotype (↓ Iba1, ↑ Arg^+^/Iba1^+^, ↑ CD206^+^/Iba1^+^, ↑ Arg^+^/Iba1^+^, ↓ iNOS^+^/Iba1^+^, ↓ microglia clustering and activate plaque-associated microglia [147, 154, 162, 163, 179, 189, 194] • Modulate astrocytes phenotype (↓ GFAP) [146, 147, 151, 159, 163, 167, 168, 179, 184] • ↓ pro-inflammatory cytokines (IL-1ß, IL-6, TNF-α and TGF-ß), ↑ anti-inflammatory cytokines (IL-4 and IL-10) [147, 149, 151, 159, 170, 176, 177, 181, 187, 189] • ↓ inflammatory mediators (TREM1, DAP12, caspase-1) [186] **Vascular damage and brain glucose metabolism** • Prevent Aß deposition in cerebral vessels. [189, 202] • Improve brain glucose metabolism and uptake (↑ GLUT1 and GLUT3) [149, 152, 179] • ↓ CSF glucose levels to control values [171] • ↑ brain glucose levels [187] • Alleviate cerebral acidosis [182] **Brain signaling pathways** • ↑ insulin signaling (activate IR/PI3K/AKT/GSK3ß pathway) [148, 164, 170, 171, 179, 183, 184, 187, 191, 194, 196, 197] • ↑ IRS2 expression [168] • ↓ GSK3ß expression [179, 181]. • ↓ mTOR signaling (↑ AMPK phosphorylation, ↓ S6K and p65 NFκB, ↓ mTOR phosphorylation,) [146, 147, 155, 163, 165, 184, 194, 197] • ↓ JNK activation [190] • ↑ atypical PKC ζ/λ phosphorylation [194] • ↓ DPP4 activity in the hippocampus. [164] • Stimulate the PKA/CREB pathway and NMDA receptors (↑PKA catalytic subunit α, NMDAR1 and CREB phosphorylation) [164] • ↓ apoptosis (↓ Bax and caspase-3, ↑ Bcl2) [148, 184] • Stimulate chaperone-mediated autophagy (↑ Hsc70 phosphorylation) [146] • Promotes autophagy (↑ beclin 1, LC3II/LC3I, ATG5 and MAP1-LC3B, ↓ p62) [163, 188] • Mitigate endoplasmic reticulum stress (↓ p-ERK, p-EIF-2α, ATF4 and CHOP) and ↓ kinases calpain 1, Cdk5 and p25 [160] • Blocks Grp78 induction and promotes the unfolded protein response [152] • ↓ phosphorylation of FGFR 1c and its co-receptor ß-klotho [166] • ↓ PARP levels [184] • ↓ brain aging pathways (↓ DNA methylation, immune response and complement activation, ↑ neuronal protection) [162]
**Sulphonylureas**	Stimulate insulin release from pancreatic β-cells	Negligible [294]	**AD neuropathological hallmarks and markers** • ↓ Aß levels (Aß42, amyloid plaques) [185, 230] • ↓ amyloidogenic pathway (↓ ß-secretase) [185] • ↓ tau phosphorylation [101] • ↑ cholinergic activity (↓ AChE activity) [185] **Oxidative stress and neuroinflammation** • ↓ Oxidative stress markers (MDA and NO) [185] • Modulate microglia phenotype (↓ Iba1) [230] • ↓ pro-inflammatory cytokines (IL-1ß, IL-6, TNF-α) [230, 232] • ↓ p65 NFκB phosphorylation [230] **Brain signaling pathways** • ↑ brain insulin signaling (↑ AKT and IRS1 phosphorylation) [187]
**SGLT2 inhibitors**	Reduce renal glucose reabsorption, lowers blood glucose	High [295]	**AD neuropathological hallmarks and markers** • ↓ Aß levels (soluble Aß40, amyloid plaques) [208, 211] • ↓ tau phosphorylation [208, 209] • ↓ brain atrophy and spontaneous hemorrhage [211] • ↑ neuronal density [209, 211] • ↑ Neurotrophic and synaptic-related factors (BDNF and synapsin-1) [204, 210] • ↑ cholinergic activity (restore AChE activity) [193, 204] • ↑ dopamine and serotonin levels [193] **Oxidative stress and neuroinflammation** • ↓ oxidative stress markers (H_2_O_2_, 4-HNE, 8-OHdG, MDA, NADPH oxidase subunits gp91 and p67, and matrix metalloproteinases) [204, 207, 210] • ↑ total antioxidant capacity (↑ glutathione reductase, glutathione peroxidase and Nrf2) [207] • Enhance mitochondrial function (↑ ATP production, complex I activity and mitochondrial membrane potential) [204, 207] • ↓ microglia accumulation near amyloid plaques [211] • ↓ TXNIP/NF-κB/NLRP3 inflammasome signaling [207] • ↓ pro-inflammatory cytokines (IL-1ß, IL-6, TNF-α) [205, 207] • ↓ gliosis (↓ GFAP^+^ astrocytes and Iba1^+^ microglia) [205] **Vascular damage** • Improve cerebrovascular function (restore cerebral blood flow autoregulation) [209] • ↓ ROS and AGEs in middle cerebral arteries [209] • Reverse BBB leakage [209] **Brain signaling pathways** • Improve brain insulin signaling (↑ AKT phosphorylation, FoxO1 translocation into cytosol) [204, 205, 208] • ↓ mTOR signaling (↓ S6 kinase phosphorylation) [205] • ↓ GSK3ß activation [204] • Modulate SIRT1/HMGB1 pathway (↑ SIRT1, ↓ HMGB1) [207] • ↑ GLP1 levels in CSF [206] • ↑ autophagy (↑ beclin-1 and LC3-II, ↓ p62 SQSTM1) [207] • Suppress neuronal apoptosis (↓ Bax and cleaved caspase-3, ↑ Bcl2) [204, 207]
**DPP4 inhibitors**	Increase GLP1 levels, improving insulin secretion and action	Minimal [296]	**AD neuropathological hallmarks and markers** • ↓ Aß levels (AB42, amyloid plaques) [159, 208, 212–216, 218–222, 225] • ↓ APP expression; ↑ IDE expression [208, 224] • ↓ tau phosphorylation [159, 208, 217–221, 228] • ↓ neurofilament H/M phosphorylation [218] • ↑ neurotrophic factors (BDNF and NGF) and neurogenesis (doublecortin) [164] • ↑ neuronal and synaptic density and markers (↑ PSD95, MAP2, synaptophysin, SAP97, synapsin-1, SP11) [159, 164, 216, 222] • Enhance synaptic plasticity (↑AMPA receptor trafficking And NMDA receptor 1 phosphorylation) [164, 213] • Restores cholinergic activity (↑ Ach levels, ↓ AChE activity) [159, 216, 222] • ↑ glutamate levels [159] **Oxidative stress and neuroinflammation** • ↓ Oxidative stress (↓ MDA, TBARS, lipid peroxidation, nitrotyrosine and nitrite, NAD(P)H oxidase subunits; ↑ glutathione, GSH, catalase activity and SOD, Nrf2 and HO-1) [159, 212, 214, 220–223, 225, 226, 228] • ↓ pro-inflammatory cytokines (IL-1ß, IL-6, TNF-α) and inflammatory mediators (CCL-5, CCL-7, CCL-12 and CXCL10) [215, 216, 226] • Modulate astrocytes phenotype (↓ GFAP) [159, 219, 226] • Modulate microglia phenotype (↓ Iba1) [223] **Brain signaling pathways** • GLP1 signaling (↑ GLP1 and GLP1 receptor levels) [212, 213, 216, 218–222] • Anti-apoptotic effects (↓ caspase-3 and Bax/Bcl2 ratio) [225, 234] • Activate the PI3K/AKT/ERK2 pathway [164, 218, 225, 226, 234] • ↓ JNK1/2 activation [218] • ↓ GSK3ß activation [215–218] • Inactivate the STAT3/JAK2 pathway [225] • Prevent insulin resistance (↓ IRS phosphorylation, ↑ IR and AKT phosphorylation) [216] • ↓ hippocampal hyperinsulinemia [215] • ↓ DPP4 activity in the hippocampus [164] • Stimulate the PKA/CREB pathway (↑PKA catalytic subunit α and CREB phosphorylation) [164] • ↑ adiponectin receptor 1 [227] • ↑ CREB phosphorylation [226] • ↓ FOXO1 and ↑ klotho [225]
**Insulin**	Reduces blood glucose levels by facilitating cellular uptake	High [297]	**AD neuropathological hallmarks and markers** • ↓ Aß levels (AB40 and AB42, soluble APPß, oligomers and amyloid plaques) [10, 234–238, 248, 249, 260, 261, 272, 274, 279]. • Promotes non-amyloidogenic APP processing (↓ BACE1 and ↑ X11α) [260, 279] • ↑ IDE expression [260, 261] • ↓ tau phosphorylation [66, 239, 248, 262, 273, 277] • Protects against neurodegeneration (↑ NeuN^+^ cells, restores MBP and PLP levels, prevents brain atrophy) [238, 240, 255, 263, 264, 271] • Promotes synaptic plasticity (↑ synapsin 1, synaptophysin, PSD95, BDNF and NR2A) [236, 241, 249, 260–263, 265, 275] • ↑ neurogenesis (doublecortin levels) [234, 260–263] • Potentiates LTP and LTD responses [243, 250, 257, 275] • Boosts neuronal activity and connectivity (↑ c-Fos expression, glutamate levels and AMPAR and NMDAR activities) [252, 254] • ↑ cholinergic activity: ↑ Ach receptors and ↓ AChE activity [265] • Prevents NMDA receptor overactivation [275] • Restores calcium homeostasis (↓ Ca^2+^-dependent afterhyperpolarization, modulates calcium networks) [254, 256] Oxidative stress and neuroinflammation • Modulate microglia phenotype (↑ P2yr12 and Cx3cr1, ↓ Iba1 and CD68) [244, 249, 260, 262, 263] • Modulate astrocytes phenotype (↓ GFAP) [260, 262, 263] • ↓ Oxidative stress (↓ 3-nitrotyrosine, iNOS, COX-2, MDA, ROS; ↑Nrf2 and glutathione) [248, 260, 265] • ↓ pro-inflammatory cytokines (IL-1ß, TNF-α, IL-10, RANTES, CXCL10 and *Ccl3*) [239, 241, 260] • ↓NF-κB activation [260] • ↑ mitochondrial activity and ATP production [265] Vascular damage and brain glucose metabolism • ↓ insulin-induced phosphorylation of IRß in microvessels [251] • ↓ RAGE levels in brain capillaries. [258] • ↓ GLUT4 overexpression [252] • Improve glucose metabolism [263] • ↑ cerebral blood flow [265] **Brain signaling pathways** • Enhances insulin signaling (↑ IR activation, total and phosphorylated AKT, ↓ IRS1 phosphorylation) [66, 234, 236, 239, 245, 248–250, 258, 260, 261, 267] • ↑ GLUT3 expression [236] • ↑ CSF insulin levels [66] • Prevents JNK and AMPK overactivation [234, 252] • ↑ CREB phosphorylation [265] • ↑ rictor and sestrin-3 (Foxo1 target genes) [244] • ↓ apoptosis (↓ cleaved caspase-3 levels, caspase-3 activity, Bax/Bcl2 ratio) [236, 240, 260] • ↑ ERK1/2 and P38 MAPK activation [240, 267] Alters the expression of 113 aging-related genes, many linked to cytokines and cell adhesion molecules [268]
**Amylin and amylin analogs**	Slow gastric emptying, suppress glucagon release, promote satiety	High [298]	**AD neuropathological hallmarks and markers** • ↓ Aß levels (AB40 and AB42, amyloid plaques, full-length APP) [171, 280–284] • Alter APP processing. [↑ BACE1] [282] • ↓ Aß transporter LRP1 in lipid rafts [285] • ↓ tau phosphorylation [171, 280, 284, 286] • Restore synaptic function (↑ LTP) [287] **Oxidative stress and neuroinflammation** • ↓ Oxidative stress (↑ SOD and glutathione peroxidase) [282] • Correct abnormal mitochondrial function-related genes expression [286] • Modulate microglia phenotype (↓ microglia number and *Cd68*) [281, 284, 286] • ↓ pro-inflammatory cytokines (IFN-γ) [284] • ↑ phosphorylation of NFκB inhibitor alpha (IκBα) [281] **Brain signaling pathways** • Normalize the expression of 975 dysregulated genes related to neuroinflammation, transcription regulation, energy homeostasis and cellular transport processes [286]. • ↓ p25 and p35 [281] • ↓ CSF glucose levels [171] • Enhance insulin signaling (↑ IR phosphorylation and PI3K) [171]

##### Effect of antidiabetic drugs on AD neuropathological hallmarks and markers

 GLP1 analogs have demonstrated protective effects in amyloid pathology models. Exenatide reduces Aß42 levels [10] and amyloid plaque area in the hippocampus [11] in AD transgenic models, and similarly exendin-4 decreases amyloid plaque load and soluble Aß levels in the cortex and hippocampus in APP/PS1 mice and Aß1-42 injected rats [12, 13]. Although, liraglutide showed no effect in one study [14], it consistently decreases Aß plaques and oligomers in the cortex and hippocampus from APP/PS1, APP^NL−G−F^ And 5xFAD mice [15–24]. Other GLP1 agonists, such as lixenatide and Val8GLP1, a protease-resistant form of GLP1, dual GLP1/GIP (gastric inhibitory polypeptide) agonists (DA-JC4, DA-JC1 and DA5-CH), the dual GLP1/glucagon agonist (D-Ser2)oxyntomodulin and a triple GLP1/GIP/glucagon triagonist, also reduce cerebral Aß plaque load in APP/PS1 mice [16, 21, 25–29]. Semaglutide effects are conflicting: a study reported that it reduces amyloid plaques, Aß40 and Aß42 levels, while also reducing tau protein concentration, in the hippocampus from APP/PS1 mice [30], whereas other work did not find such effects neither in APP/PS1 nor in 5xFAD mice [31]. Besides reducing Aß plaques, the GLP1 agonist NLY01 also decreases BACE1 (ß-secretase 1) expression in 5xFAD mice [32]. Additionally, liraglutide and DA5-CH attenuate tau hyperphosphorylation in the hippocampus from AD mice [24, 25, 33] and non-human primates [34], and exenatide and other GLP1 agonists increase cortical and hippocampal synaptic density in both genetic and Aß-induced AD models [11, 19–22, 33, 35, 36]. Specifically, exenatide elevates post-synaptic density protein 95 (PSD95) And synaptophysin levels, improving synaptic structure in 5xFAD mice [11], whereas NLY01 increases microtubule-associated protein 2 (MAP2), PSD95 and brain-derived neurotrophic factor (BDNF) in the same model [32]. In this line, GLP1 receptor agonists enhance synaptic function and long-term potentiation (LTP). Exendin-4 improves cholinergic activity by augmenting acetylcholine (ACh) levels and choline acetyltransferase (ChAT) activity, while decreasing acetylcholinesterase (AChE) activity in Aß1-42 injected rats [12]. Exenatide increases synaptic vesicles and reduces synaptic cleft width [11], liraglutide restores chemical synapses [33] and Val8GLP1 protects against Aß-induced synaptic dysfunction [37]. Treatments with liraglutide, lixenatide, Val8GLP1, DA4-JC, DA5-CH and (D-Ser2) oxyntomodulin and the GLP1/GIP/glucagon triagonist protect LTP [19–22, 25–27, 38–42] and promote neurogenesis and neuroblast differentiation in the hippocampus and subventricular zone [16, 17, 19–22, 28, 43].

The effects of TZD on amyloid pathology are controversial. Pioglitazone reduces soluble Aß40 and Aß42, non-fibrillar Aß levels and amyloid plaques in APP transgenic mice [83–86], though other studies found no effect in similar models [87–89]. Interestingly, pioglitazone-loaded nanocarriers show greater efficacy than freely delivered pioglitazone against amyloid pathology in APP/PS1 mice [90]. Pioglitazone modulates amyloid processing by downregulating APP [84] and decreasing α-secretase and BACE1 levels in different models of APP transgenic mice [85, 91]. Rosiglitazone has shown clearer benefits, reducing Aß oligomers, Aß40 and Aß42 levels [92–95] and preventing Aß plaque formation in the hippocampus and cortex in APP transgenic mice [92, 95]. Other PPAR-γ agonists Like 15d-PGJ2, pan-PPAR activator GFT1803 and PPARα/γ agonists DSP-8658 and N15 also reduce Aß levels in these regions [96–99], with N15 also promoting the non-amyloidogenic pathway, increasing α-secretase levels while reducing BACE1 and presenilin-1 levels [97]. Both pioglitazone and rosiglitazone enhance Aß transport by increasing ATP-binding cassette A1 expression [86, 92, 100], with pioglitazone also upregulating ApoE levels [86, 100] and its lipidation index [92, 100] in APP transgenic models. In addition, pioglitazone and DSP-8658 boost Aß phagocytosis by glial cells [86, 99], and rosiglitazone promotes CD36 expression, aiding Aß internalization by microglia [92]. Furthermore, rosiglitazone prevents insulin-degrading enzyme (IDE) downregulation in the hippocampus and enhances its activity in the frontal cortex [94], and similarly GFT-1803 enhances Aß degradation through IDE overexpression [96]. Furthermore, both drugs reduce tau hyperphosphorylation [92, 101] and improve synaptic plasticity, increasing synaptic proteins synaptophysin, vesicle-associate membrane proteins and PSD95 [95], while restoring BDNF levels [102] in both transgenic and Aß-injected AD models. Likewise, PPARα/γ agonist N15 prevents neuronal apoptosis, increasing the number of hippocampal neurons, and promotes the expression of the neurotrophic factor *Bdnf* [97]. Additionally, pioglitazone prevents synaptic spines elimination [103] and improves cholinergic activation by increasing ChAT [87] and decreasing AChE overactivity [102] in the hippocampus from APP transgenic mice and Aß-administered rats [91]. Pioglitazone also rescues LTP impairment in hippocampal slices [104] and long-term depression (LTD) induction in cerebellar slices [105] from APP/PS1 mice. Rosiglitazone improves synaptic function, presynaptic hyperactivity and short-term plasticity on hippocampal slices [106], and it also reverts excitability in dentate gyrus neurons in Tg2576 mice [107].

The effects of metformin on amyloid pathology are mixed. In APP/PS1 mice, metformin reduces APP [146] and BACE1 protein expression [147], amyloid plaques, soluble Aß40 and Aß42 and insoluble Aß42 levels [146–149], likely through IDE overexpression [149]. However, in 5xFAD mice, metformin increases Aß plaque deposition and soluble and insoluble Aß42 levels in the frontal cortex [150]. Conversely, metformin reduces neuronal injury and tau hyperphosphorylation in rats receiving Aß42 [148], prevents neuronal death in the hippocampus [151] and promotes adult neurogenesis by upregulating neurotrophic factors *Bdnf* and *Ngf*, and the synapse-related protein synaptophysin in APP/PS1 mice [147, 149]. In addition, metformin decreases AChE activity in Aß injected rats [151].

On the other hand, the SU glibenclamide reduces Aß deposition in 5xFAD mice [230] and tau hyperphosphorylation in the hippocampus from Aß-injected rats [101]. DPP4i like alogliptin, linagliptin and sitagliptin consistently decrease Aß42 levels, amyloid plaques and neuronal damage in the same region in APP/PS1 and Aß-administered animals [212–216]. Vildagliptin also decreases tau phosphorylation, prevents neuronal damage and upregulates PSD95 and synaptophysin levels in the hippocampus from intracerebroventricular Aß-injected rats [217]. Accordingly, sitagliptin increases synaptic density in the CA1 region, augments BDNF levels and enhances synaptic plasticity by improving AMPA receptors trafficking in APP/PS1 mice [213], while linagliptin attenuates AChE overactivity in Aß-injected rats [216].

Insulin treatment shows neuroprotective effects across different administration routes. Intranasal insulin promotes non-amyloidogenic APP processing, reducing Aß42, Aß40, soluble APPß, oligomers and Aß plaques in the cortex and hippocampus from APP/PS1, Tg2576 And 5xFAD mice, as well as in Aß-injected rats [10, 234–236]. Interestingly, co-assembly of insulin with macrocyclic amphiphiles potentiates its anti-amyloid effects [236]. Subcutaneous insulin also decreases Aß42 levels in presenilin-2 overexpressing mice [237], whereas hypodermic insulin mitigates Aß40 accumulation and amyloid deposits in Aß-intrahippocampal injected rats [238]. Moreover, insulin reduces tau hyperphosphorylation and protects against hippocampal neurodegeneration in Aß-injected rats [238–240], while it increases doublecortin and *Bdnf* levels in APP/PS1 mice [234, 241]. In addition, intranasal insulin preserves Nissl bodies And mitigates neuronal apoptosis, while also upregulating synaptic proteins NMDA receptor 2A, PSD95 And synaptophysin in the hippocampus from 5xFAD mice [236]. Additionally, ex vivo insulin maintains neuronal morphology and facilitates GABA-activated currents in young though not in older hippocampal slices from APP mice [242], and rescues Aß42-induced LTP impairment independently of insulin receptor (IR) activation [243].

Amylin and its analogs also show benefits in amyloid pathology models. Amylin and pramlintide reduce amyloid plaques, lower Aß40 and Aß42 levels and decrease full-length APP. Amylin additionally increases Aß42 levels in cerebrospinal fluid (CSF), while pramlintide boosts α-secretase and BACE1 expression in APP transgenic mice [280–284]. Both treatments decrease low density Lipoprotein receptor-related protein 1 (LRP1), which mediates Aß transport across the blood–brain barrier (BBB), in lipid rafts from transgenic APP Swedish/Indiana mice [285]. Amylin also decreases tau phosphorylation in the cortex, hippocampus and thalamus from APP transgenic mice [280, 281, 283, 284, 286]. While effects on PSD95 expression seem to be mixed [280, 285], both drugs restore synaptic function and partially reverse Aß42-induced LTP depression in hippocampal slices [287].

##### Effect of antidiabetic drugs on oxidative stress and neuroinflammation

Hypoglycemic agents Have been widely studied for their effects on oxidative stress And neuroinflammation. Exenatide improves mitochondrial function in 5xFAD mice, enhancing mitochondrial morphology and increasing ATP levels and complex I activity, while normalizing mitochondrial dynamics – decreasing the fission gene dynamin-related protein 1 *(Drp1)* And increasing the fusion genes optic atrophy type 1 (*Opa1*) And mitofusin 2 (*Mfn2*) [11]. Similarly, exendin-4 protects against Aß42-induced mitochondrial dysfunction by improving mitochondrial bioenergetics [12]. Exenatide also reduces oxidative damage by lowering malondialdehyde (MDA) levels and increasing superoxide dismutase (SOD) activity in the hippocampus from 5xFAD mice [11], though it does not affect mitochondrial COX activity in PSEN1-knockin mice [44]. Similarly, Liraglutide improves mitochondrial function by increasing phosphorylated dynamin-related protein 1, mitofusin 2 and OPA1, mitigating reactive oxygen species (ROS) overproduction And boosting ATP production in 5xFAD mice [36], and a GLP1/GIP/glucagon triagonist also reduces oxidative stress Markers such as 4-HNE (4-hydroxy-2-nonenal) And 8-OHdG (8-hydroxy-2'-deoxyguanosine) in APP/PS1 mice [28]. While one study found no effect of liraglutide on microglial phenotype in APP/PS1 mice [24], others report that Liraglutide modulates glial phenotypes, reducing ionized calcium binding protein 1 (Iba1)^+^ microglia and astrocytes glial fibrillar acidic protein (GFAP) overexpression in the cortex and hippocampus from APP/PS1 and APP^NL−G−F^ mice [15, 19–23]. Results for semaglutide are mixed: one study reported that it modulates glial phenotypes [30], whereas other did not find such effect in 5xFAD neither APP/PS1 mice [31]. Conversely, dual GLP1 agonists and triagonists have been shown to modulate microglia and astrocyte phenotypes [16, 27, 28, 35]. Furthermore, GLP1 agonist NLY01 reverses reactive astrocyte markers overexpression in the hippocampus, including the genes *Lcn2*, *Osmr* and *Ggta1*, and the proteins GFAP and C3 in 5xFAD mice [32], whereas DA-JC4 has been shown to decrease TNF-α and IL-1ß levels in APP/PS1 mice [27].

The TZD pioglitazone restores mitochondrial enzyme activities and reduces MDA and nitrite levels in Aß-injected rats [102], with region-specific effects: it decreases complex I activity in the hippocampus but increases it in the cortex from PS1 knock-in mice. Pioglitazone counteracts oxidative stress by upregulating ROS scavenging enzymes SOD1 and peroxiredoxin-5, enhancing antioxidant defenses (glutathione, SOD and catalase activities) and downregulating ROS promoting proteins in APP transgenic mice [84, 108]. In addition, pioglitazone lowers cyclooxygenase 2 (COX-2) and inducible nitric oxide synthase (iNOS) expression in PS1 knock-in mice [109]. However, pioglitazone shows mixed effects on neuroinflammation, decreasing astrocyte and microglia activation in the hippocampus and frontal cortex from APP transgenic mice in several studies [83, 85, 87, 88], while other noted microglia colocalization with amyloid plaques in the cortex from APP/PS1 mice [100]. Some studies have described that pioglitazone may reduce Iba1 expression [100, 110]. and increase Ym1, Fizz1 and Arg1 markers, alongside lowering interleukin (IL)−1ß, IL-6, tumor necrosis factor alpha (TNF-α) and caspase-3 levels [86, 100, 102], whereas others report no effect on microglia activation [83, 89]. Conversely, rosiglitazone modulates microglia phenotype, promoting a shift to a ramified morphology expressing Ym1 and Fizz1 markers in APP transgenic mice [92], thereby reducing TNF-α, COX-2, IL-1ß and interferon gamma (IFN-γ) levels [92, 111], and decreasing CD11b^+^ microglia colocalization with Aß plaques [93] in both transgenic and Aß-injected AD models. Furthermore, rosiglitazone switches astrocytes and microglia to a surveillant state in APP/PS1 mice [95], whereas ciglitazone modulates only microglia phenotype in APP transgenic mice [110]. Other PPAR agonists, including 15d-PGJ2 and GFT1803, also reduce neuroinflammation in the cortex and hippocampus [96, 98], with 15d-PGJ2 decreasing inflammatory markers – monocyte chemoattractant protein-1, TNF-α, IL-1ß and IL-6 levels [98] –, and GFT1803 reducing microglia surrounding Aß plaques [96].

The biguanide metformin decreases AD-induced oxidative stress and neuroinflammation. Specifically, metformin lowers MDA, nitrites, protein carbonyl and ROS levels, while boosting SOD activity in amyloid pathology models [149, 151]. In addition, metformin reduces microglia and astrocytes activation in the cortex and hippocampus and reactive glial cells around amyloid plaques in APP/PS1 mice [146, 147], as well as GFAP expression and Aß-injected rats [151]. Concomitantly, metformin decreases pro-inflammatory cytokines (IL-1ß, IL-6 and TNF-α), while increasing the anti-inflammatory cytokines IL-4 and IL-10 [147, 149, 151].

Other antidiabetics, such as the SU glibenclamide, reduce microglia activation and cytokine levels (TNF-α, IL-1β, and IL-6), and decrease p65 NFκB phosphorylation in 5xFAD mice [230]. DPP4i alogliptin, sitagliptin and linagliptin enhance antioxidant defenses (glutathione, catalase, SOD, Nrf2 and heme oxigenase-1) and reduce MDA, thiobarbituric acid reactive substances (TBARS), nitrotyrosine and nitrite levels in APP/PS1 and Aß-injected models, while also lowering IL-1ß, IL-6 and TNF-α levels in APP/PS1 and Aß injected models [212, 214–216]. Intranasal insulin in APP/PS1 mice upregulates microglial homeostatic markers *P2yr12* and *Cx3cr1* in the hippocampus [244] and decreases *Il1b* and *Tnfa* expression in intrahippocampal Aß-injected rats [239], whereas intraperitoneal insulin decreases *Ccl3* expression in APP/PS1 mice [241]. Amylin and pramlintide also affect oxidative stress and neuroinflammation and amylin corrects abnormal mitochondrial function-related gene expression in the cortex [286] And counteracts microglia activation in the cortex, hippocampus And thalamus in 5xFAD mice [281, 284, 286], while pramlintide shows region-dependent effects: it decreases heme oxygenase-1 expression in the cortex but increases it in the hippocampus, raises SOD in the hippocampus and increases glutathione peroxidase in both regions in APP/PS1 mice [282]. Amylin lowers IFN-γ levels in brain and CSF [284] and increases NFκB inhibitor alpha (IκBα) phosphorylation in 5xFAD mice [281], whereas pramlintide exacerbates microglia activation in APP transgenic mice [285].

##### Effect of antidiabetic drugs on vascular damage and brain glucose metabolism

Antidiabetics role in managing blood glucose levels secondarily affects both systemic vascular health and cerebral blood flow, yet their direct effects on the brain remain underexplored. Exenatide increases brain lactate dehydrogenase (LDH) activity and levels of β-hydroxybutyric acid And lactic acid, suggesting higher brain glucose levels And glycolytic intermediates such as glucose 6-phosphate And 3-phosphoglyceric acid in PS1 *knock-in* mice [44]. Furthermore, liraglutide improves cerebral microvasculature integrity by reducing microaneurysms and cerebral extravasations in APP/PS1 mice [45]. Pioglitazone normalizes cerebrovascular reactivity and dilatory responses, boosts nitric oxide (NO) production and restores neurovascular and neurometabolic coupling, cerebral blood flow and glucose uptake in response to neuronal activity, while downregulating SOD2 in cerebral blood vessels in APP transgenic mice [87, 88]. Proteomic analysis in APP/J20 mice revealed that pioglitazone reduces cerebrovascular Aß toxicity, normalizing levels of cerebrovascular proteins related to vascular tone [108]. Moreover, pioglitazone also restores several brain metabolites in the cortex and cerebellum from APP/PS1 mice [84]. Similarly, the PPAR-γ agonist 15d-PGJ2 improves brain glucose metabolism by upregulating glucose transporter GLUT4 in the same mouse model [98]. Metformin increases neuronal GLUT1 expression in *Drosophila* flies overexpressing Aß42, thereby enhancing glucose uptake [152], and it similarly improves brain glucose metabolism in APP/PS1 mice [149]. Finally, insulin promotes GLUT3 expression in the hippocampus from 5xFAD mice [236].

##### Effect of antidiabetic drugs on brain signaling pathways

Hypoglycemic agents affect brain pathways through various mechanisms. GLP1 Analogs regulate insulin pathway genes including IR, glycogen synthase kinase 3ß (GSK3ß), IR substrate (IRS)1/2, AKT1/3, insulin-like growth factor-1 receptor and Wnt/ß-catenin components [10, 13, 18, 29], activate the phosphatidylinositol 3-kinase (PI3K)/AKT pathway, inactivate GSK3ß [12, 25, 33, 35], and reverse IRS1 phosphorylation in different models overexpressing Aß [13, 23]. Liraglutide also normalizes neuronal IR localization near Aß plaques in APP/PS1 mice [23], prevents Aß-induced IR loss [34] and boosts cAMP levels and PKA phosphorylation and activity in both transgenic and Aß-injected models [15, 36, 39]. Furthermore, liraglutide shifts astrocytic aquaporin-4 localization around brain blood vessels, contributing to an increased water flux in the glymphatic system in APP^NL−G−F^ mice [15]. Remarkably, exendin-4 reduces pro-apoptotic Bax And cleaved caspase 3 levels, increases anti-apoptotic Bcl2 [46] and alleviates calcium overload by upregulating calcium/calmodulin-dependent protein kinase II in Aß injected rats hippocampal neurons [47], while antagonizing Aß42-induced toxicity by activating the cAMP/PKA/CREB pathway [40]. NLY01 and liraglutide increase GLP1 receptor expression in the hippocampus from 5xFAD and Aß42-injected mice [32, 33], while DA-CH3 reduces endoplasmic reticulum stress and restores autophagy-related proteins in APP/PS1 mice [35]. Supporting the diverse pathways modulated by GLP1 receptor agonists, semaglutide treatment in APP/PS1 mice has been shown to partially reverse several AD-related gene expression changes [30].

Pioglitazone affects cerebral LDH activity in a sex-dependent manner in PS1 knock-in mice [109]. It also reduces p25 and p35 protein expression in APP/PS1 mice [105], and restores Wnt and ß-catenin levels in Aß40 injected mice [91]. Similarly, rosiglitazone increases Dvl-3 and ß-catenin levels, reducing GSK3ß activation in APP/PS1 mice [95], and prevents glucocorticoid receptors downregulation in APP/J20 mice [112]. Rosiglitazone also upregulates several SNARE proteins involved in vesicle dynamics regulation [106], stimulates the extracellular regulated kinase (ERK)/mitogen-activated protein kinase (MAPK) pathway and increases PPAR-γ activity in Tg2576 mice [106, 113]. The PPAR-γ agonist 15d-PGJ2 activates PPAR-γ coactivator 1α/GLUT4 signaling and reduces IRS1 phosphorylation in APP/PS1 mice [98], while the dual PPARα/γ agonist N15 activates the expression of PPARα and PPARγ mRNA [97].

Metformin activates 5’ adenosine monophosphate-activated protein kinase (AMPK), reducing phosphorylation of mammalian target of rapamycin (mTOR), S6K and p65 NFκB, and stimulates chaperone-mediated autophagy in APP/PS1 mice [146, 147]. Metformin also blocks Grp78 induction, promotes the unfolded protein response in Aß-overexpressing *Drosophila* flies [152], reduces Aß-induced apoptosis and activates the AKT/ERK pathway in Aß-injected rats [148, 151]. DPP4i sitagliptin increases brain GLP1 levels in APP/PS1 mice [212, 213], while vildagliptin shows anti-apoptotic effects and activates the AKT pathway in Aß-injected rats [217]. Alogliptin and linagliptin attenuate Aß-induced GSK3ß activation and prevent insulin resistance markers, such as IRS phosphorylation at Ser307, in Aß-injected rats [215, 216].

Insulin administration, whether intranasal or via reverse microdialysis, enhances insulin signaling by increasing IRß, total and phosphorylated AKT, and reducing IRS1 hyperphosphorylation in APP/PS1 mice and Aß-oligomer injected rats [234, 239, 245]. In Tg2576 mice, intranasal insulin tends to normalize insulin pathway gene expression and, when combined with exenatide, significantly downregulates these genes [10]. Conversely, insulin increases GSK3ß activation [244], prevents JNK pathway overactivation [234] and upregulates rictor and sestrin-3 in APP/PS1 mice [244]. Moreover, insulin reduces Aß-induced apoptosis by reducing the Bax/Bcl2 ratio And lowering caspase 3 in neurons [236], and it also decreases ERK and P38 MAPK activation [240]. Conversely, amylin and pramlintide increase cleaved caspase-3 in APP transgenic mice [285], And amylin normalizes the expression of 975 dysregulated genes related to neuroinflammation, energy homeostasis and cellular transport processes [286], while reducing p25 and p35 and increasing IκBα levels in 5xFAD mice [281].

##### Effect of antidiabetic drugs on cognitive performance

Hypoglycemic agents improve cognitive functions in amyloid pathology models. Exenatide, exendin-4 and NLY01 consistently improve spatial learning and memory in the Morris Water Maze (MWM) [11, 12, 32, 40, 41, 46, 48, 78, 110] and in the Y-maze tests [12, 32], with exendin-4 also ameliorating circadian rhythm [48], while semaglutide improves cognitive performance in the Barnes maze, MWM and active avoidance tasks [30]. Similarly, liraglutide prevents memory impairment in MWM, object location and novel object recognition (NOR) test, Y-maze and fear conditioning tests in Aß-injected and amyloid pathology transgenic models [15, 19–22, 33, 34], although it does not significantly improve passive avoidance in 5xFAD mice [18]. However, GLP1 medications may be more effective in young animals. A study evaluating the GLP1 receptor agonists semaglutide and tirzepatide in APP/PS1 And 5xFAD mice found no cognitive improvement in the NOR or MWM tests at 12 months of age, while a modest improvement in the NOR test was observed in 5xFAD mice at 6 months of age [31]. Remarkably, intracerebroventricular liraglutide before Aß injection prevents spatial learning and memory impairments in the MWM test [39]. Dual GLP1 agonists, along with GLP1/GIP/glucagon triagonist, enhance cognitive flexibility and spatial memory in the MWM and the Y-maze tests in APP/PS1 mice [25, 27–29, 35].

TZD administration in amyloid models shows mixed effects on cognitive performance. Pioglitazone generally improves spatial learning and long-term memory in the MWM, including Aß-injected, PS1 *knock-in* and APP/PS1 models [91, 99, 101, 102, 104, 109], while some discrepancies have been reported in other APP transgenic mice [87, 88]. Notably, pioglitazone’s positive effects in the MWM were observed specifically in female PS1 *knock-in* mice [109]. Pioglitazone also improves cerebellar function, assessed by the rotarod test, in APP/PS1 mice [105], and enhances performance in the Y-maze, passive avoidance and contextual fear conditioning tests in Aß40-injected and APP/PS1 mice [91, 100]. Remarkably, nanocarrier-encapsulated pioglitazone is more effective than free-administered pioglitazone in the NOR in APP/PS1 mice [90]. Likewise, rosiglitazone prevents cognitive impairment and restores memory in the NOR and MWM tasks in both Aß oligomers-injected and APP/PS1 mice [92, 93, 95, 111, 112], and Similar effects Have been reported in the 8-arm radial maze test [94] and in the fear conditioning test with Tg2576 mice [113, 114], though some studies suggest acute administration only improves navigational learning and memory in this model [115]. Broad-spectrum PPAR agonists also attenuate spatial learning and memory impairments in the MWM test in APP/PS1 mice [96, 98, 99], and similar improvements in the same test have been reported for dual PPAR α/γ agonist N15 in Aß-injected mice [97].

Metformin ameliorates spatial learning and memory in the MWM, NOR, elevated plus maze, radial arm maze and Y-maze tests in APP/PS1 mice and Aß-injected rats [146–149, 151], and enhances climbing ability in Aß-overexpressing flies [152]. Metformin also shows sex-specific effects in APP transgenic mice, improving MWM performance in females but worsening it in males [153]. Though one study found no improvement in MWM spatial learning in 5xFAD mice [230], glibenclamide enhances cognitive performance in the MWM, passive avoidance and elevated plus maze tests in Aß25-35-administered rats [101, 231], and alleviates depression and anxiety-related symptoms in the sucrose preference, the forced swimming and the light–dark box tests [231].

DPP4i like vildagliptin, alogliptin, linagliptin and sitagliptin improve Aß-impaired spatial learning and memory in the MWM test in APP transgenic and Aß-administered models [213–217], with sitagliptin also enhancing fear conditioning performance in APP/PS1 mice [212]. Insulin generally improves MWM and Y-maze test performance in Aß-injected models And 5xFAD and Tg2576 transgenic mice [10, 236, 238–240, 246, 247]. In addition, insulin also reduces anxiety-related behaviors and improves performance in the reversal MWM test in APP/PS1 mice [234]. However, intranasal insulin does not seem to improve cognitive performance in other tasks such as the T-maze or fear conditioning tests [247]. On the other hand, intraperitoneal insulin does not seem to ameliorate cognitive impairment in Aß25-35-injected rats in the MWM neither in APP/PS1 in the Barnes maze [241, 253]. Likewise, a single post-learning dose of intranasal insulin impairs spatial memory retention in the MWM in the same model [244]. Noteworthy, combined intranasal insulin and exenatide shows synergistic effects in Tg2576 mice [10]. Amylin and pramlintide improve learning and memory in MWM and Y-maze tests in APP/PS1, 5xFAD, Tg2576 and Dutch mice [280, 282–284], with amylin also enhancing social performance assessed by nest construction task in 5xFAD mice [281], though one study found no MWM improvement in APP TgSwDI mice [285].

#### Transgenic mice reproducing tau pathology or amyloid and tau pathologies concomitantly

Studies on antidiabetic treatments often use mouse models that replicate tauopathies by overexpressing human tau mutations (P301L and P301S), linked To neurodegeneration, as well as 3xTg-AD and tau-injected APP/PS1 mice that mimic both amyloid and tau pathologies.

##### Effect of antidiabetic drugs on AD neuropathological hallmarks and markers

Various antidiabetic treatments have yielded diverse effects on AD-related pathology in tauopathy or mixed amyloid-tau models. While exenatide does not seem to reduce Aβ levels or tau phosphorylation in the hippocampus from 3xTg-AD mice [44], semaglutide reduces Aß plaque deposition [49] and liraglutide decreases Aβ42 and Aβ40 brain levels And attenuates tau phosphorylation in tau And 3xTg-AD mice [50–52]. Additionally, liraglutide prevents neurodegeneration and decreases neurofilament-H and -M phosphorylation in the same model [50]. Likewise, NLY01 increases MAP2 And BDNF levels in 3xTg-AD mice [32], and a GLP1/GIP/glucagon triagonist elevates synaptophysin and PSD95 expression, while reducing pyramidal neuron over-excitability in 3xTg-AD hippocampal slices [53].

Other studies have revealed that pioglitazone reduces intracellular Aβ in the CA1 region, decreases soluble Aβ and downregulates *Apoe* and *Apba2* expression, involved in lipid shuttling and Aß processing, in 3xTg-AD mice [116]. However, pioglitazone’s effects on tau pathology are mixed: it does not affect tau phosphorylation in P301S mice [117] but decreases tau hyperphosphorylation in 3xTg-AD animals [116]. Pioglitazone also downregulates genes related to glutamatergic neurotransmission, including *Slc1a1*, *Slc1a2*, *Grm3*, *Grid2*, and *Grin1*, while upregulates *Grid1* and *Grin3a*, improving short- and long-term plasticity [116].

Metformin’s effects are inconsistent. In APP/PS1 mice injected with tau aggregates, metformin reduces amyloid plaques in the CA3 region and limits tau aggregates spreading [154]. Similarly, in P301S mice, it reduces tau hyperphosphorylation in the cortex and hippocampus and limits tau aggregates transmission [155, 156], while it also seems to increase insoluble tau species and inclusions [155]. Likewise, chronic metformin treatment on 3xTg-AD mice exacerbates amyloid pathology, increasing both amyloid plaques and Aß oligomers, and promotes tau phosphorylation and GSK3α and GSK3ß expression [157]. Metformin´s effects on protein phosphatase 2 A expression are conflicting, with one study showing an increase [155] and another showing no effect on tau-related kinases or phosphatases [156]. Additionally, metformin has been found to disrupt synaptic structure by reducing synaptophysin expression in tauopathy models [155].

DPP4i sitagliptin, saxagliptin, and linagliptin reduce tau phosphorylation and Aβ42 levels in the cortex and CA3 area from 3xTg-AD mice [218, 219], with linagliptin also decreasing Aβ deposits [219]. Likewise, sitagliptin and saxagliptin alleviate neurodegeneration by increasing synaptic markers and decreasing neurofilament H/M phosphorylation in 3xTg-AD mice [218].

Intranasal insulin has revealed no effects on full-length APP expression neither Aß dodecamers in the frontal cortex or hippocampus, but it decreases Aß oligomers in the hippocampus [248] and Aß40 in the forebrain of 3xTg-AD mice [249]. However, its impact on tau pathology is conflicting, with one study showing reduced tau phosphorylation in the hippocampus from 3xTg-AD mice [248], while another found no effect in the same mice [249]. Nevertheless, insulin promotes synaptic plasticity by increasing synaptic markers (synapsin 1, synaptophysin, and PSD95) in the olfactory bulb And prefrontal cortex from 3xTg-AD mice [249], and enhances insulin responsiveness and LTD in hippocampal slices from Tau22 transgenic mice [250]. Finally, amylin has been shown To reduce tau phosphorylation in 3xTg-AD mice [284].

##### Effect of antidiabetic drugs on oxidative stress, neuroinflammation and brain glucose metabolism

Exenatide does not affect mitochondrial COX activity in 3xTg-AD mice [44], while Liraglutide improves mitochondrial function by reducing 8-OHdG, nitrite, carbonyl groups and TBARS levels, and decreases *Fis1* and *Opa* overexpression in the same model [51]. Semaglutide modulates microglial phenotype in 3xTg-AD mice, lowering the *Cd86/Cd206* ratio and shifting cytokine expression by reducing *Il1b* and *Tnfa* while increasing *Il4* and *Il10* in the cortex and hippocampus [49]. Similarly, NLY01 reduces hippocampal inflammatory markers *Tnfa*, *C1q*, *C3*, *Il1b*, *Ifng*, *Il6*, and *Gfap* in 3xTg-AD mice [32]. TZD pioglitazone downregulates pro-inflammatory genes (*Tnfaip2*, *Tnfrsf1a*, *Il1rap*, *C1ql3*, *C1ql2* and *NFκB*) and upregulates genes reducing cytokine signaling (*Socs2*, *Socs5*, and *Lpin1*) in 3xTg-AD mice [116], but it does not seem to modulate microglia phenotype in P301S tau mice [117]. Exenatide And pioglitazone do not affect LDH activity or Anaerobic glucose catabolism in 3xTg-AD mice [44, 109], and liraglutide does not alter brain GLUT1/and GLUT4 expression or glucose levels in 3xTg-AD mice [51]. However, liraglutide prevents glucose-6-phosphate dehydrogenase activity decline and reduces cerebral pyruvate and lactate levels in early AD [51].

On the other Hand, metformin activates plaque-associated microglia, promoting tau aggregates phagocytosis And reducing tau pathology spread in 3xTg-AD mice [154]. DPP4i linagliptin decreases GFAP levels in the same model [219]. Insulin also shows neuroprotective effects: intracarotid insulin attenuates phosphorylation of IRß in microvessels in 3xTg-AD mice [251], while intranasal insulin decreases 3-nitrotyrosine levels in the cortex and hippocampus [248], reduces microglial number and CD68 expression in the same model [249]. Similarly, amylin reduces microglia number and *Cd68* gene expression, though protein levels remain unchanged in 3xTg-AD mice [284].

##### Effect of antidiabetic drugs on brain signaling pathways

Studies on hypoglycemic agents on signaling pathways in tau pathology models are limited. Liraglutide and NLY01 increase GLP1 and GLP1 receptor levels, respectively [32, 51], whereas semaglutide does not affect GLP1 receptor expression in 3xTg-AD mice [49]. Liraglutide also normalizes ERK1/2 and JNK1/2 activities [50], decreases PKA activity and reduces levels of the autophagy-related protein p62 in the same model [51]. Similarly, metformin decreases p62 in microglia in 3xTg-AD mice [154], inactivates mTOR pathway [155], reducing mTORC1 protein levels [156], and activates AMPK signaling and caspase-3 in P301S mice [155], upregulating the expression of AMPKα1 subunit in 3xTg-AD mice [157]. Additionally, DPP4i elevate GLP1 and GLP1 receptor levels, enhancing GLP1 signaling in models of amyloid and tau pathologies [218, 219], and activate PI3K, AKT and ERK1/2, while inactivating GSK3ß and JNK1/2 [218]. Finally, intranasal insulin increases IR activation, activates the PI3K/AKT/mTOR pathway, reduces IRS1 phosphorylation and enhances ERK1/2 activation in 3xTg-AD mice [248, 249].

##### Effect of antidiabetic drugs on cognitive performance

Studies on GLP1 analogs show varied Outcomes. Exenatide does not improve spatial memory in the MWM test in 3xTg-AD mice [44], while liraglutide and semaglutide enhance cognitive performance in MWM and Y-maze tests in the same model [49–51], and liraglutide also reduces clasping in P301L mice [52]. NLY01 improves spatial memory but not learning in the MWM test [32], and a GLP1/GIP/Glucagon triagonist enhances both working and reference memory in the radial arm Maze in 3xTg-AD mice [53]. Similarly, pioglitazone exhibits mixed effects, improving learning in the active avoidance test [116] but not cognition in the MWM or NOR in 3xTg-AD mice [109]. Metformin treatment worsens cognitive decline And impairs associative learning in 3xTg-AD mice [157] and increases hyperactivity and exacerbates hindlimb atrophy in P301S mice [155], but attenuates learning and memory deficits in the MWM test in the same model [156]. On the other hand, DPP4i improve spatial learning And memory in the MWM And Y-maze tests in 3xTg-AD mice [218, 219]. Likewise, intranasal insulin enhances short-term memory in the MWM and NOR [248], And amylin improves learning And memory in the MWM and Y-maze tests in 3xTg-AD mice [284].

### Preclinical models of sporadic AD and AD-like dementia

**Fig. 3:**
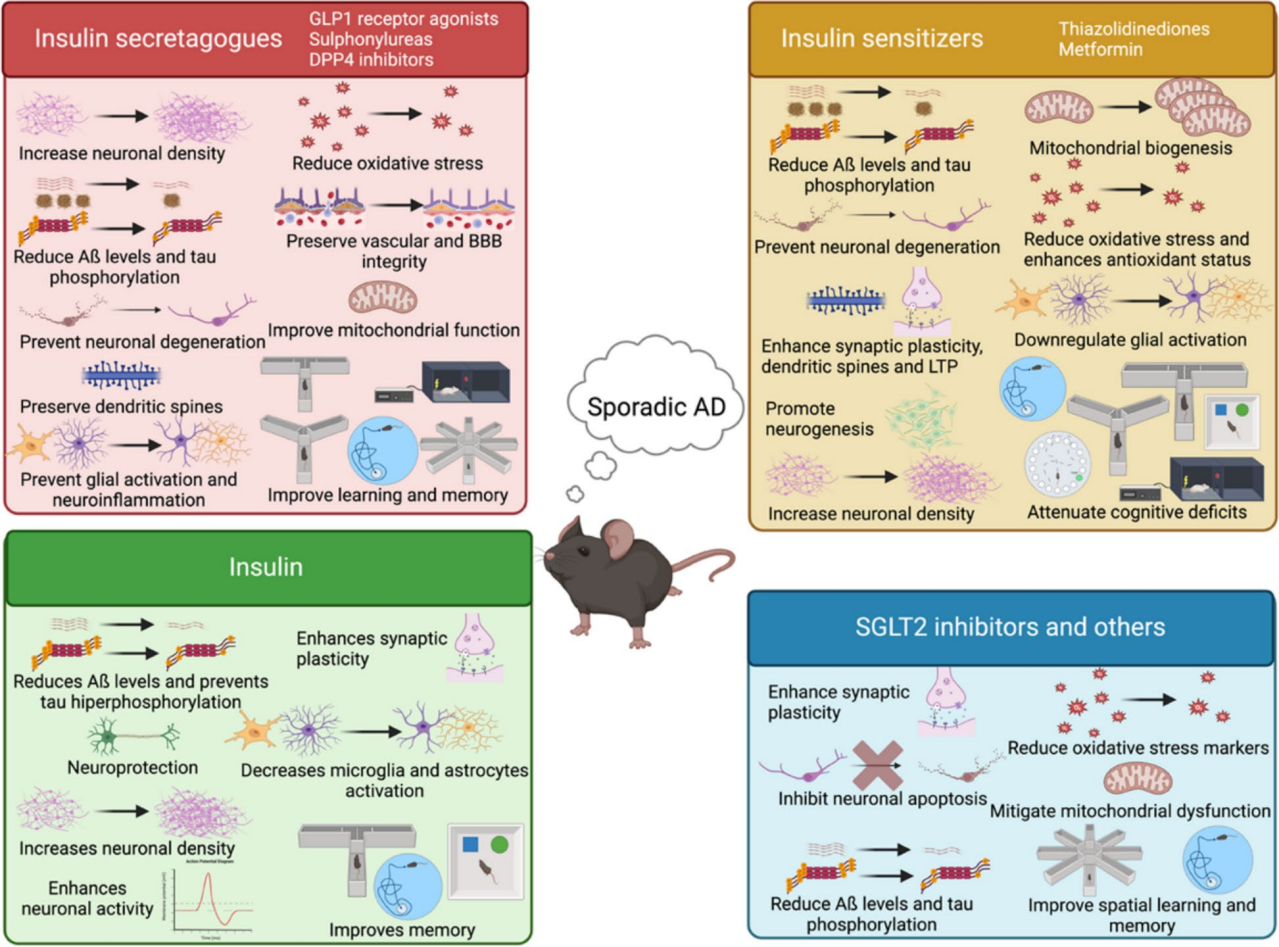
Beneficial effects of antidiabetic drugs as disease-modifying therapies for dementia on preclinical models of sporadic AD or AD-like dementia. Created with BioRender.com

Most AD cases are sporadic, with less than 3% linked to genetic mutations. Research has focused on animal models replicating sporadic AD, including: i) age-related models, using aged wildtype mice/rats or SAMP8 mice to mimic AD-like dementia; ii) models based on genetic risk factors like *ApoE* ε4, linked to both early- and late-onset AD in genome-wide association studies (GWAS); and iii) models involving environmental factors, heavy metal exposure, chemicals like streptozotocin (STZ) administered intracerebroventricularly, scopolamine, aluminum chloride (AlCl_3_), and hyperhomocysteinemia. Key findings are summarized in Fig. 3 and bibliographic references are compiled in Table 1.

#### Effect of antidiabetic drugs on AD neuropathological hallmarks and markers

##### Age-related models

Liraglutide treatment in SAMP8 mice increases neuron density in the hippocampal CA1 region but does not prevent brain atrophy [54]. The TZD pioglitazone reduces Aß40 and Aß42 levels in the hippocampus of SAMP8 mice [118], counteracts age-related downregulation of LRP1 receptors in endothelial cells [118], and restores calcium homeostasis in aged rats [119]. Rosiglitazone attenuates brain damage as shown by magnetic resonance imaging (MRI) and enhances LTP in aged rats [120]. Other antidiabetic treatments, including metformin and saxagliptin, prevent Aß accumulation, amyloid plaques deposition, tau hyperphosphorylation, and neuronal degeneration in cortical and hippocampal regions, while partially restoring ACh and glutamate levels in aged animals and SAMP8 mice [158–162]. The effects of metformin alone are controversial: one study did not find increased neurogenesis neither synaptogenesis in aged mice [163], while other work in aged monkeys found increased neural precursor activity and synaptic connectivity in the hippocampus, along with preserved cortical thickness in the frontal lobe [162]. On the other hand, both linagliptin and metformin upregulate neurotrophic factors (*Bdnf*, *Ngf* and *Nmdar1*) and synaptic markers (spinophilin, synaptophysin and *Psd95*) in the hippocampus from aged mice, and concomitantly promote synaptic plasticity by upregulating doublecortin and MAP2 [164].

Insulin’s neuroprotective effects on age-related dementia depend on the administration route. Acute intracerebroventricular insulin partially reverses neural connectivity decline in aged rats [254], while long-term intranasal insulin increases NeuN^+^ cells in the hippocampus of SAMP8 mice [255]. Additionally, short-action insulin reduces calcium-dependent hippocampal after hyperpolarization ex vivo [254], and acute insulin modulates calcium networks in the somatosensory cortex, decreasing neuronal excitability and increasing connectivity and synchronicity [256].

##### *ApoE* transgenic mice

Different TZDs show beneficial effects: pioglitazone rescues dendritic spine deficits in the entorhinal cortex of *ApoE* ε4 mice [121], while rosiglitazone promotes mitochondrial biogenesis in the frontal cortex [122] and decreases malic enzyme and *Ldh* mRNA expression in both wildtype and *ApoE knockout* mice, suggesting reduced pyruvate to lactate conversion [122]. Conversely, metformin shows mixed effects. Metformin increases tau phosphorylation in aged ‘humanized’ *ApoE* ε3 and *ApoE* ε4 transgenic mice [165], yet exacerbates neurodegeneration, tau phosphorylation and neuroinflammation in ApoE *knockout* mice [166]. However, metformin upregulates synaptic proteins in a genotype-dependent manner, increasing vesicular GLUT1, synaptophysin, PSD95, GluR1, NR2A and NR2B in hippocampal synaptosomes from aged *ApoE* ε3 mice, but only post-synaptic proteins in aged *ApoE* ε4 mice [165]. Insulin´s effects also depend on *ApoE* allele: in APPxApoE ε3 mice, insulin potentiates LTP ex vivo in hippocampal slices, but this effect is absent in APPxApoE ε4 mice [257], where intravenous insulin may worsen cortical tau phosphorylation [258].

##### Other sporadic-AD models

Antidiabetic treatments in sporadic AD models have varied effects on neuropathology. Liraglutide reduces APP expression and phosphorylation, BACE1 and PSEN1, and increases α-secretase in hyperhomocysteinemia and AlCl_3_-induced AD [55, 56]. However, liraglutide’s effects on Aß deposition are mixed, showing no reduction in intraneuronal Aß levels in STZ-treated mice [18], but decreasing hippocampal Aß40 levels in hyperhomocysteinemia-induced AD [56]. GLP1 agonists DA-JC4, dulaglutide, exenatide and liraglutide reduce tau hyperphosphorylation in hyperhomocysteinemia and STZ-induced sporadic AD [56–60], with dulaglutide and liraglutide also preventing neurofilament H/M hyperphosphorylation [59, 60]. Exenatide, exendin-4 and liraglutide protect against hippocampal neuronal loss, preserving dendritic spines by upregulating synapsin-1 and glutamatergic receptors [55–57, 59–62]. Specifically, exendin-4 protects against neuronal apoptosis by reducing cleaved caspase 3 levels [62]; while liraglutide enhances neurotransmission by increasing adrenaline, noradrenaline and dopamine levels in AlCl_3_-treated rats [55].

Among TZD, rosiglitazone reduces Aß40 and Aß42 levels in the hippocampus of STZ-induced AD rats and increases LRP1 and ATP-binding cassette A1 levels, aiding Aß clearance [123]. Similarly, the PPAR-δ/γ agonist T3D-959 decreases Aß42 levels in the temporal lobe, mitigates tau hyperphosphorylation, reduces neuronal loss and white matter thinning, and improves synaptic function and synaptophysin expression in the hippocampus from STZ-treated rats [124]. Rosiglitazone also upregulates neurotrophic factors (*Creb*, *Bdnf*, *Gdnf* and *Ngf*), (*Creb*, *Bdnf*, *Gdnf* and *Ngf*), as well as PPAR-γ in mice receiving intracerebroventricular STZ [291, 292]. On the other hand, pioglitazone protects the cholinergic system by augmenting ACh levels and restoring AChE and ChAT activity in various sporadic AD models [125–127].

Metformin has multiple benefits in sporadic AD, reducing Aß levels and tau hyperphosphorylation [167–172], increasing neuronal density and alleviating hippocampal histopathological alterations, preserving Nissl bodies in various models [167, 168, 172–177]. Metformin also enhances neurogenesis, upregulating *Dcx* and *Ki67* transcription in the hippocampus from AlCl_3_-induced AD mice [173], and upregulates neurotrophic factors CREB (cAMP-response element binding protein), BDNF and glial cell line-derived neurotrophic factor in scopolamine-induced AD rats [177, 178]. Additionally, metformin increases AMPK levels and promotes its phosphorylation [172, 178], boosts synaptic function by increasing synapsin-1 [179] and dopamine [167], and improves cholinergic function by several ways – increasing Ach levels and ChAT activity, while decreasing AchE and butyrylcholinesterase activities [167, 175, 177, 179–181] in different sporadic AD models.

SGLT2i ertugliflozin and canagliflozin inhibit neuronal apoptosis, upregulate BNDF and synapsin-1, and restore AChE activity [203, 204] in STZ-treated rats. Additionally, canagliflozin decreases hippocampal tau protein and amyloid pathology by reducing both Aß and BACE-1 levels, while increasing IDE protein [203]. DPP4i like vildagliptin, saxagliptin and linagliptin prevent neuronal degeneration and decrease Aß42 levels in the hippocampus [220–222]. Saxagliptin and vildagliptin also reduce tau hyperphosphorylation in the cortex and hippocampus [220, 221], while linagliptin reduces AChE activity in STZ-induced AD [222].

Insulin treatment in STZ-administered rats decreases amyloid plaque deposition and Aß42 levels in the cortex and hippocampus [259–261], increases IDE expression [260, 261] and prevents tau hyperphosphorylation, likely via GSK3ß, ERK1/2 and CaMKII inactivation [259, 262]. In addition, insulin prevents hippocampal atrophy neuronal loss, increasing neuronal density, doublecortin, PSD95 and BDNF levels [259–265]. Insulin also boosts neuronal activity by increasing c-Fos expression, glutamate levels and AMPAR and NMDAR activities in STZ-treated rats [252], but it does not restore cholinergic transmission in AlCl_3_-treated rats [266]. Interestingly, intranasal insulin restores neuronal network dynamics [259] and restores cholinergic activity in the cortex and hippocampus from STZ-induced AD rats [265]. Likewise, the amylin analog pramlintide prevents hippocampal Aß accumulation and tau hyperphosphorylation in STZ-induced AD [171].

#### Effect of antidiabetic drugs on oxidative stress, neuroinflammation, vascular damage and brain glucose metabolism

##### Age-related models

The effects of GLP1 analogs on neuroinflammation and vascular damage are understudied. Exenatide reduces immune-related gene expression (*Apoe*, *Ccl4*, *Ccl6*, *Cd9*, *Cd52* and *Timp2*) in microglia [63, 64] and promotes a rejuvenated phenotype in astrocytes in aged mice [63]. Exenatide also enhances vascular integrity, reversing age-related transcriptomic changes in smooth muscle and endothelial cells [63, 64], preventing cortical vascular leakage and improving BBB function, and upregulating energy metabolism genes that typically decline with aging [64].

In contrast, the TZD rosiglitazone shows minimal impact on glial cells and does not downregulate microglial disease-associated markers [120] or IL-1ß and protein carbonyl levels in aged rats [128]. On the other hand, insulin reduces GFAP overexpression and lowers RANTES, CXCL10 and TNF-α levels in aged rats [120]. Metformin treatment in aged mice modulates glial phenotypes, reducing microglial clustering in the hippocampus and promoting CD206 expression, and similarly decreasing the occurrence of reactive astrocytes, resulting in decreased levels of IL-1ß, IL-6 and TNF-α [163]. Similarly, in aged monkeys metformin reduced microglial accumulation in the hippocampus [162].

Accordingly, other studies reported that metformin and saxagliptin prevent hippocampal astrogliosis and reduce receptor for advanced glycation end products (RAGE) expression [159], and metformin and linagliptin alone or in combination modulate microglia phenotype and decrease CCL-3 [163, 164], with the combined treatment promoting a switch in the inflammatory profile, decreasing *Il1b*, *F4/80*, iNOS expression and NF-κB activation, while upregulating *Arg1*, *Il10*, *Mgl1* and *Mgl2* expression [164]. Conversely, the effects of metformin on oxidative stress are not so clear. Metformin alone does not seem to modulate oxidative stress markers MDA and protein carbonyls [163], whereas metformin and saxagliptin have been reported to lower oxidative stress markers (MDA, NADPH oxidase and TNF-α), increase glutathione [159] and alleviate cerebral acidosis in the aging brain [182]. SGLT2i canagliflozin reduces age-associated gliosis in region and sex-specific manners: it reduces GFAP^+^ astrocytes and Iba1^+^ microglia in the hypothalamus in both males and females, but these effects are only observed in male mice in the hippocampus [205].

##### Other models of sporadic AD

Antidiabetic treatments alleviate oxidative stress and neuroinflammation in sporadic AD models. GLP1 agonists liraglutide and DA-JC4 modulate microglia and astrocyte phenotypes [18, 58], with exenatide lowering TNF-α levels in STZ-treated rats [61]. TZDs pioglitazone and rosiglitazone reduce oxidative markers (MDA, lipid peroxidation and nitrite), restore glutathione levels and promote SOD activity in STZ- and scopolamine-induced AD models [125, 126, 129, 130, 291, 292]. Pioglitazone and rosiglitazone also lower IL-6 and TNF-α cytokines in STZ-induced AD [126, 291, 292], while T3D-959 decreases several pro-inflammatory cytokines (IL-12, IFN-γ, macrophage colony-stimulating factor, monocyte chemoattractant protein-1 and RANTES), and increases anti-inflammatory cytokines (IL-10, IL-1α and IL-7) in the temporal lobe from STZ-treated rats [124]. On the other hand, metformin reduces MDA and nitrite levels and myeloperoxidase activity, while it also enhances antioxidant status, increasing glutathione, glutathione peroxidase, and catalase and glutathione transferase and SOD activities in various sporadic AD models [167, 168, 170, 175, 177, 178, 181]. Metformin also downregulates glial activation markers Iba1 and GFAP in STZ-induced AD rats [167, 168, 172, 179], while reducing the overexpression of IL-1ß, IL-6, TNF-α and TGF-ß in both scopolamine- and STZ-induced models [170, 176, 177, 181]. Furthermore, metformin reduces CSF glucose levels to control values [171] and upregulates glucose transporters (GLUT1 and GLUT3) in the cortex and hippocampus of STZ-treated rats [179].

Other antidiabetic treatments also show potential. The SU glibenclamide reduces IL-6 and TNF-α hippocampal levels in STZ-administered rats [232]. SGLT2i ertugliflozin reduces oxidative stress markers H_2_O_2_ And 4-HNE, matrix metalloproteinases and mitochondrial dysfunction in STZ-treated rats [204], whereas phloridzin does not modulate catalase activity in the same model [206]. Canagliflozin improves mitochondrial function and improves redox status in sporadic AD models, reducing MDA levels while increasing total antioxidant capacity, glutathione reductase, glutathione peroxidase and Nrf2 [203, 207]. Canagliflozin also shows anti-inflammatory activity by inactivating the TXNIP/NF-κB/NLRP3 signaling and the inflammatory cascade [203, 207]. DPP4i vildagliptin, saxagliptin and linagliptin reduce IL-1ß and TNF-α in STZ-induced AD rats [220–222], with linagliptin also lowering TBARS and nitrite levels and increasing catalase activity [222].

Insulin has shown several beneficial outcomes in sporadic AD models. It regulates oxidative-nitrosative stress markers – increasing Nrf2 and glutathione, while decreasing iNOS expression, MDA and nitrite levels and ROS production – and increases mitochondrial activity, boosting ATP production, in STZ-induced AD rats [265]. Furthermore, insulin modulates neuroinflammation by decreasing microglia and astrocytes numbers in the hippocampus, as well as Iba1 and GFAP expression in the cortex and hippocampus, inhibiting COX-2 and NF-κB activation and normalizing TNF-α and IL-10 levels in STZ-treated models [260, 262–264]. In addition, Insulin also improves glucose metabolism in the prefrontal and cingulate cortices [263], reduces GLUT4 overexpression in the cortex and hippocampus [252] and improves cerebral blood flow in STZ-administered rats [265], though it may not prevent oxidative stress in AlCl_3_-induced models [266].

#### Effect of antidiabetic drugs on signaling pathways

##### Age-related models

Research on signaling pathways affected by hypoglycemic agents in aging models remains limited. Pioglitazone unexpectedly reduces IR phosphorylation in the cortex, likely due to its peripheral effect on insulin levels [119]. Conversely, metformin and saxagliptin lower brain insulin levels while increasing phosphorylated IRs [159]. Metformin’s impact on GSK3ß activation in SAMP8 mice is debated, with conflicting effects on phosphorylation sites [158, 160]. Additionally, metformin downregulates kinases Like calpain 1, Cdk5 and p25, and mitigates endoplasmic reticulum stress [160], while increasing AMPK phosphorylation in neurons in aged mice [163]. A recent multi-omics study in aged monkeys reported that metformin treatment decelerates brain aging, decreasing DNA methylation and reverting neurons and glial cells to a more youthful state, downregulating pathways that are usually upregulated during aging, while upregulating neuroprotective genes and pathways [162]. Remarkably, both metformin and linagliptin treatments reduce hippocampal DPP4 activity, with a higher effect when administered together in old mice [164]. In addition, these drugs alone or in combination stimulate the insulin and the PKA/CREB pathways, and inactivate p38 MAPK [164].

SGLT2i canagliflozin shows sex-specific effects, improving insulin sensitivity in the hypothalamus, and concomitantly reducing mTOR signaling in the hippocampus from male aged mice [205]. Insulin’s effects on brain signaling are similarly complex: it activates AKT ex vivo in young brain slices but not aged ones [267], whereas acute insulin administration does not affect canonical insulin signaling in SAMP8 mice [268]. However, insulin indirectly activates ERK1/2 ex vivo [267], And intranasal insulin alters the expression of 113 aging-related genes in the hippocampus, many linked to cytokines and cell adhesion molecules [268].

##### *ApoE* transgenic models

Antidiabetic drugs modulate different pathways in ApoE models. Rosiglitazone regulates genes involved in mitochondrial biogenesis, increasing estrogen-stimulated related receptor alpha in *ApoE knockout* mice and decreasing PPAR-γ cofactor 1α in both *ApoE knockout* and *ApoE* ε4 mice [122]. Metformin downregulates phosphorylation of fibroblast growth factor receptor 1c and its co-receptor ß-klotho in *ApoE knockout* mice, but does not affect AMPK or mTOR pathways [166]. Besides, metformin promotes GSK3ß activity in both *ApoE* ε3 and *ApoE* ε4 aged mice, with insulin signaling activation observed only in *ApoE* ε3 mice [165]. Interestingly, metformin modulates AMPK and mTOR pathways in an *ApoE*-dependent manner, specifically in *ApoE* ε3 mice [165].

Insulin’s effects on ApoE alleles are controversial. A single insulin injection increases AKT phosphorylation only in *ApoE* ε4, but reduces RAGE levels in brain capillaries of both *ApoE* ε3 and ε4 mice [258]. In addition, hippocampal slices from ApoE ε4xAPP mice are unresponsive to insulin, whereas those from *ApoE* ε3xAPP and APP mice are responsive, suggesting a synergistic impairment of insulin signaling by *ApoE* ε4 and Aß deposition [257].

##### Other models of sporadic AD

Antidiabetic treatments targeting insulin signaling show promise in sporadic AD models. Liraglutide reduces IRS1 phosphorylation, increases GLP1 receptors And protein-tyrosine phosphatase 1B levels [56], inhibits GSK3ß [18, 58] and modulates MAPK pathways by activating ERK1 and deactivating JNK1/2 [59]. Dulaglutide restores GLP1 and GLP1 receptor levels and reactivates the PI3K/AKT/GSK3ß pathway in STZ-induced AD rats [60]. Similarly, GLP1 analogs DA-JC4 and exenatide also activate AKT and prevent GSK3ß activation in the same model [57, 58]. T3D-959 restores insulin/insulin-like growth factor 1/AKT signaling and inactivate GSK3ß in STZ-treated rats [124], an effect similarly observed with pioglitazone [131], which also reduces caspase-3 activity [126].

Metformin stimulates brain insulin signaling by enhancing IR, PI3K and AKT activation, downregulating GSK3ß expression [170, 171, 179, 181, 183], while increasing hippocampal IRS2 levels [167, 168]. Furthermore, metformin shows anti-apoptotic and anti-pyroptotic effects by reducing caspase-1 and caspase-3 activities [172], reduces mTOR signaling through activation of AMPK and enhances autophagy by increasing beclin-1, ATG5 and MAP1-LC3B, while decreasing p62 in the hippocampus [163].

SGLT2i ertugliflozin promotes AKT phosphorylation [204], while canagliflozin activates the AMPK/sirtuin-1 pathway, decreasing high mobility group box 1 protein and GSK3ß levels [203, 207]. Canagliflozin also augmented autophagy, increasing beclin-1 and LC3-II levels while decreasing p62 SQSTM1 levels, and prevented apoptotic events by decreasing caspase-3 and Bax levels [207].

On the other hand, phloridzin increases GLP1 levels in CSF [206], as observed with DPP4i [220–222]. Insulin upregulates IR expression and activates the IRS1/AKT/GSK3 pathway [260], preventing AMPK overactivation [252], and stimulates CREB phosphorylation [265]. Besides, the amylin analog pramlintide reduces CSF glucose levels and enhances insulin signaling by increasing IR phosphorylation and PI3K levels [171].

#### Effect of antidiabetic drugs on cognitive performance

##### Age-related models

Several pharmacological agents enhance cognitive function in age-related models. For instance, liraglutide improves long-term memory in the active avoidance T-maze [54], the TZD rosiglitazone boosts hippocampal-dependent memory in contextual fear conditioning [128], and pioglitazone improves learning and memory in the MWM test [118]. The biguanide metformin attenuates cognitive deficits in the MWM, Y-maze, T-maze, NOR, Barnes maze or pattern separation tests in age-related rodent models [158–161, 163] and enhances memory retention, learning ability and cognitive flexibility in aged monkeys [162]. In contrast, long-term metformin treatment in mice throughout their lifespan appears to negatively affect memory retention and discrimination learning in older age (16 months) [157].

Noteworthy, a comparative study on old mice reports that metformin has superior effects than linagliptin in short-term memory in the NOR and passive shuttle avoidance tests, while both drugs similarly improve performance in the Y-maze test, with combined treatment further improving cognition [164]. Likewise, DPP4i saxagliptin also improves spatial learning and memory in the MWM test [159]. SGLT2i canagliflozin improved exploratory activity in open field test in aged male mice [205]. Notably, acute intranasal insulin (short- or long-acting) significantly boosts spatial memory recall in the MWM [267], reference memory consolidation in the T-maze and declarative memory in the NOR [269].

##### *ApoE* transgenic models

Few studies have evaluated antidiabetic drugs’ effects on cognition in ApoE transgenic mice. Metformin improves spatial learning and memory in the MWM test in aged *ApoE* ε3 mice, but not in *ApoE* ε4 mice [165], while a single insulin dose does not alleviate cognitive deficits in *ApoE* ε4 mice in the NOR [258], whereas intranasal insulin improves MWM performance in *ApoE* ε4 but not in *ApoE* ε3 mice [278].

##### Other sporadic AD models

Antidiabetic treatments consistently improve cognition in sporadic AD models. DA-JC4 and exendin-4 enhance Y-maze performance [58, 62], while exendin-4, liraglutide, DA-JC4 and dulaglutide improve MWM performance in STZ- and hyperhomocysteinemia-induced AD models [56–60]. Exenatide and liraglutide improve passive avoidance and eight-radial arm maze tasks in STZ- and AlCl_3_-treated models, respectively [55, 61], And exendin 4 also protects discrimination memory in the NOR in STZ-induced AD [62]. The TZD pioglitazone reverses memory deficits in the autoshaping test [131] and improves learning and memory in MWM and step-through passive avoidance in STZ- and scopolamine-induced AD [125–127, 129, 130], with acute administration shortly before cognitive tests improving memory retrieval in the Y-maze and the passive avoidance tests in scopolamine-induced AD [132, 133]. Metformin benefits cognitive function in MWM, elevated plus maze, Y maze, Barnes maze, classic labyrinth, passive avoidance and NOR tests in sporadic AD models [167–176, 178–181, 183].

The SU glibenclamide similarly Y-maze and MWM performance in STZ-induced AD [232]. Likewise, the SGLT2i ertugliflozin and canagliflozin improve spatial learning and memory in MWM, Y-maze and NOR in the in sporadic AD models [203, 204, 207], while phloridzin shows no effect [206]. DPP4i saxagliptin, vildagliptin and linagliptin improve cognition in several tests, including the radial arm maze, hole board, and MWM in STZ-induced AD [220–222]. Intraperitoneal insulin does not ameliorate AlCl_3_-induced cognitive dysfunction in the MWM test [266], but intranasal and intracerebroventricular insulin significantly enhance MWM performance in STZ-induced AD [259–262, 264, 265, 270]. Likewise, pramlintide improves learning and memory in MWM and NOR in STZ-treated rats [171].

### Preclinical models of metabolic impairment

#### Type 1 diabetes (T1D) models

**Fig. 4:**
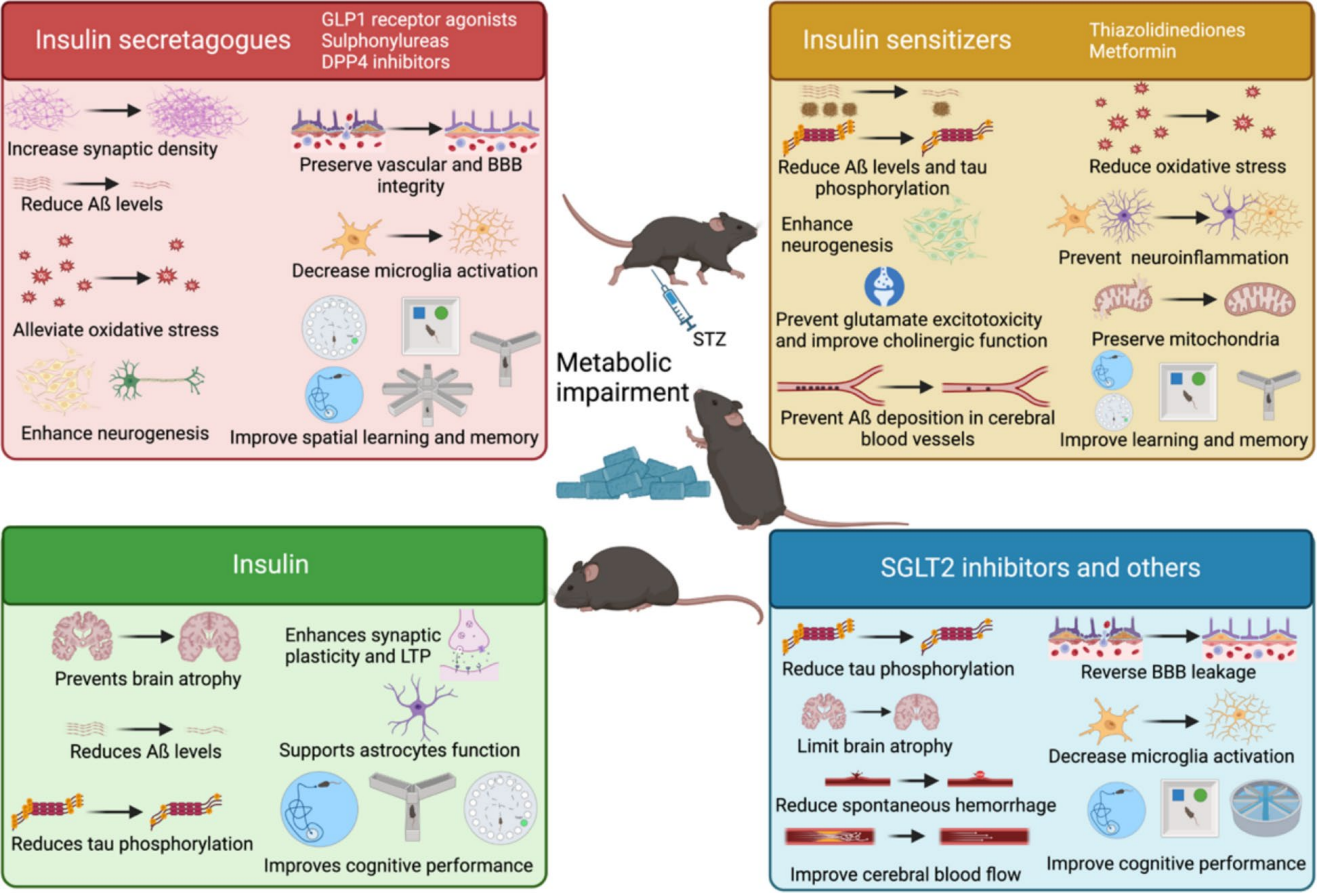
Protective effects of antidiabetic drugs as disease-modifying therapies for dementia on preclinical models of metabolic impairment. Created with BioRender.com

Chronic hyperglycemia in T1D is linked to cognitive impairment, including memory, attention and executive function deficits. Chemicals like STZ and alloxan are commonly used to induce experimental T1D in murids via intraperitoneal administration, and the effects of hypoglycemic agents at brain level have been largely assessed. Major findings are summarized in Fig. 4, with references in Table 1.

##### Effects of antidiabetic drugs on AD neuropathological hallmarks and markers

Several antidiabetic drugs show promise in reducing AD neuropathological markers in T1D models. Liraglutide alleviates hippocampal ultrastructural damage and synapse injuries in STZ-induced T1D, lengthening post-synaptic density [65]. Metformin offers neuroprotection by reducing Aß burden in the parietal cortex [184], lowering Aß42 through decreased ß-secretase expression [185], and preventing tau hyperphosphorylation in the cortex and hippocampus from STZ-induced T1D models [184–186]. Additionally, metformin mitigates NMDA receptor 2A-mediated excitotoxicity and reduces AChE overexpression [184, 185]. The SU glibenclamide reduces ß-secretase levels, Aß42 accumulation and AChE activity [185]. Insulin therapy also shows significant benefits: subcutaneous insulin prevents brain atrophy [271], reduces hippocampal Aß42 [272] and partially prevents tau hyperphosphorylation [273]. Similarly, intranasal insulin lowers Aß levels in the hippocampus of STZ-induced T1D mice [274]. Furthermore, subcutaneous insulin administration restores myelin basic protein and proteolipid protein [271], prevents NMDA receptor overactivation [275] and enhances synaptic plasticity and LTP in the CA1 region [275].

##### Effects of antidiabetic drugs on oxidative stress and neuroinflammation

Antidiabetic drugs are increasingly recognized for their role in modulating oxidative stress and inflammation in the T1D brain. Liraglutide alleviates oxidative stress in the hippocampus from STZ-induced T1D mice by decreasing MDA levels and increasing SOD activity [65]. Similarly, metformin prevents astrocyte activation and reduces inflammatory markers (*Trem1, Dap12*, *Il1b* and *Tnfa)* and oxidative stress markers (MDA, NO, iNOS and COX-2) in various brain regions in T1D models [184–186]. The SU glibenclamide also lowers MDA and NO levels, enhancing neuroprotection in STZ-induced T1D [185]. Linagliptin, a DPP4i, decreases oxidative stress by reducing lipid peroxidation and downregulating NAD(P)H oxidase subunits, concomitantly reducing microglia activation and TNF-α levels in the cortex [223]. In contrast, insulin therapy does not seem to reverse hippocampal oxidative stress [272], but increases GFAP levels, which are decreased in T1D rats, supporting astrocyte function [271].

##### Effects of antidiabetic drugs on brain signaling pathways and cognitive performance

Antidiabetic drugs modulate signaling pathways in T1D-induced neurodegeneration through various mechanisms. Liraglutide restores the PI3K/AKT pathway, decreases GSK3ß levels, reduces apoptosis and improves spatial learning and memory in the MWM test in STZ-induced T1D mice [65]. Similarly, metformin enhances brain insulin resistance by increasing AKT phosphorylation, restores AMPK activation, downregulates the ERK cascade and improves cognitive performance in the Barnes maze [184]. Both the SU glibenclamide and the DPP4i linagliptin improve cognitive performance in the radial arm water maze in both alloxan- and STZ-induced T1D [223, 233].

Insulin’s effects on neurodegeneration depend on the administration route. While subcutaneous insulin does not affect hippocampal insulin levels in STZ-induced T1D rats [272], delivery via mini osmotic pumps increases CSF insulin in the same model [271]. Noteworthy, ex vivo insulin administration enhances IR and GSK3ß phosphorylation in T1D-hippocampal slices [276]. Interestingly, subcutaneous insulin implants restore brain insulin sensitivity in STZ-induced T1D mice [273, 276]. Consistently, insulin improves cognitive performance in the MWM, Y-maze and Barnes maze test in STZ-induced T1D models [272, 273, 275].

#### Prediabetes (PreDM) and type 2 diabetes (T2D)

Several models have been developed to study PreDM and T2D preclinically, including: i) hypercaloric diets, namely—high-fat diet (HFD), high-fat/high-sugar diet, high fructose diet, and high-fat/high-sugar/high-salt diet –; ii) monogenic obesity models, such as the ob/ob and db/db mice and Zucker diabetic fatty rats; iii) chemically-induced T2D models, including HFD/STZ and nicotinamide/STZ combined treatments; and iv) spontaneous T2D strains, including Otsuka Long Evans Tokushima Fatty, Goto-Kakizaki And type 2 diabetic nephropathy rats. Key findings are summarized in Fig. 4 and references are compiled in Table 1.

##### Effect of antidiabetic drugs on AD neuropathological hallmarks and markers

GLP1 receptor agonists, including exenatide, exendin-4 and liraglutide consistently show neuroprotective effects against tau hyperphosphorylation in T2D models [66–70, 79]. Exendin-4 and liraglutide also promote neurogenesis in the dentate gyrus of PreDM and T2D mice [71], exenatide increases *Ngf* expression in the hippocampus of T2D mice [72], and liraglutide and Val8GLP1 restore hippocampal LTP, disrupted by HFD [73, 74]. Interestingly, Val8GLP produces better effects when combined with metformin, which per se does not enhance LTP [73]. Additionally, semaglutide increases brain serotonin levels and glutamatergic receptors, as well as neuronal density, in HFD-fed mice [75]. Beneficial effects have also been reported for TZDs. Pioglitazone inhibits the amyloidogenic pathway, reducing BACE1, PSEN1, Aß42 levels and amyloid plaques [136–138], while increasing IDE and *Pparg* expression [136, 138] in PreDM and T2D models. However, pioglitazone’s effect on neuronal health in fructose-induced T2D is mixed, with inconsistent findings on CA1 protection, but it consistently improves cholinergic activity [137, 139]. Similarly, rosiglitazone increases BDNF, GluR1 and NR2A levels in the hippocampus, improving LTP ex vivo in db/db mice [140] and insulin-induced LTD in PreDM rats [141].

Metformin and glimepiride prevent amyloid pathology by decreasing APP and BACE1 expression, increasing IDE levels, and reducing Aß42 deposition, also reversing tau hyperphosphorylation in both PreDM and T2D models [187–190]. Furthermore, both drugs improve cholinergic function by reducing AChE activity [187, 191]. Noteworthy, metformin effects vary between PreDM and T2D: it promotes BDNF expression and prevents synaptophysin loss in T2D but not PreDM models [190–192]. However, metformin prevents diet-induced neuronal loss and hippocampal volume reduction [188, 189], and increases cortical dopamine levels [193]. In HFD-fed mice, metformin restores hippocampal neurogenesis and enhances neuronal maturation [194]. SGLT2i also show neuroprotective effects, with empagliflozin and luseogliflozin reducing tau hyperphosphorylation, brain atrophy and spontaneous hemorrhage, while increasing neuronal density [208, 209, 211], and empagliflozin additionally increasing BDNF [210], whereas canagliflozin decreases AChE activity and increases cortical dopamine and serotonin levels in T2D rats [193].

DPP4i sitagliptin reduces Aß levels, restores IDE expression and decreases tau hyperphosphorylation in HFD/STZ induced-T2D mice [208], but exacerbates tau phosphorylation in Otsuka Long Evans Tokushima Fatty rats [224]. Vildagliptin decreases Aß42 levels in the hippocampus of PreDM rats [225], and alogliptin restores hippocampal neuronal density in Zucker diabetic fatty rats [226]. Notably, insulin’s efficacy varies by administration method: intranasal insulin reduces tau hyperphosphorylation in HFD/STZ mice [66, 277], whereas subcutaneous insulin has minimal effects [66, 67, 277].

##### Effects of antidiabetic drugs on oxidative stress, neuroinflammation and vascular damage

Few studies have explored the impact of antidiabetic drugs on brain vascular damage. GLP1 analog exendin-4 preserves vascular integrity by reducing vessel tortuosity, branching and density, and restoring occludin expression in the BBB of STZ/HFD mice [76]. Liraglutide reduces hemorrhage in the cortex and hippocampus from db/db mice [79]. Semaglutide treatment on HFD-fed mice shows anti-inflammatory effects in the hippocampus – inactivating the TLR4/MyD88/NF-κB pathway, while reducing IL-1ß, IL-6, TNF-α and nytrotirosin levels –, concomitantly modulating microglia and astrocytes phenotypes [75]. Similarly, Val8GLP1 reduces hippocampal astrocyte activation And 8-OHdG levels in the same model [73]. TZD pioglitazone enhances antioxidant defenses in T2D models by increasing SOD, catalase and glutathione peroxidase activities, while reducing ROS, TBARS, myeloperoxidase, lipid peroxidation and protein oxidation, and inhibiting NADPH oxidase [137, 139, 142]. Pioglitazone also mitigates neuroinflammation by decreasing NFκB activation, and reducing TNF-α, advanced glycation end products (AGEs) and RAGE levels [142].

The SGLT2i luseogliflozin improves cerebrovascular function, reduces ROS and AGEs in middle cerebral arteries, restores cerebral blood flow autoregulation and reverses BBB leakage in the neocortex and hippocampus from T2DN rats [209]. Similarly, empagliflozin ameliorated oxidative stress by reducing NADPH oxidase subunits gp91 and p67, superoxide And 8-OHdG levels in db/db mice [210]. On the other hand, metformin prevents diet-induced amyloid ß deposition in cerebral vessels [189] exhibits strong antioxidant effects in PreDM and T2D models by reducing ROS production, preventing mitochondrial damage [195], and enhances mitochondrial ATP production and coupling [196]. Metformin also reduces oxidative stress markers (MDA, TBARS, and NO, lipid peroxidation and xanthine-oxidase activity) [187, 195, 197], and improves brain antioxidant capacity by increasing SOD activity in the hypothalamus and hippocampus from T2D rats [197], while boosting citrate synthase and COX activities in HFD-fed mice [196]. Furthermore, metformin shifts microglial phenotype, prevents chronic microglial activation in the hippocampus [194], increases arginase-1 and decreases iNOS and TNF-α expression in the motor and sensory cortex [189]. Metformin also decreases levels of IL-1ß, IL-6 and TNF-α, as well as AGES, RAGE and iNOS [187, 189]. The SU glimepiride and glibenclamide similarly reduce oxidative stress and neuroinflammatory markers in T2D rats [187, 232]. Likewise, SGLT2i empagliflozin reduces cortical and hippocampal microglia burden in db/db mice [211], whereas DPP4i vildagliptin and alogliptin reduce inflammation and oxidative stress. Specifically, vildagliptin counteracts high-fat/high-sugar diet-induced TNF-α and FOXO1 elevation [225], and alogliptin decreases hippocampal GFAP expression, inactivates the NFκB pathway, restores oxidative balance and reduces inflammatory mediators (CCL-5, CCL-7, CCL-12 and CXCL10) in Zucker diabetic fatty rats [226].

##### Effect of antidiabetic drugs on brain signaling pathways

Antidiabetic drugs impact brain insulin signaling in T2D models. Liraglutide elevates CSF insulin levels in db/db mice [67], similar to exendin-4, which also augments insulin levels in various T2D models [66, 69]. Val8GLP1 and metformin individual treatments upregulate *mTOR*, *Vegf*, *Sirt1*, and *Ntrk2* expression, while combined treatment produces further increases and also upregulates GLP1 receptor expression [73]. In line with this, semaglutide increases hippocampal GLP1 receptor levels in the hippocampus [75] and GLP1 analogs increase GLP1 levels in CSF and cortex in various T2D models [68, 70, 72, 77]. Liraglutide counteracts age-related AKT downregulation and GSK3ß overactivation [67], while exendin-4 restores the AKT/GSK3ß pathway by increasing IRS1 phosphorylation and reactivating the Wnt/ß-catenin/NeuroD1 pathway [66, 68–70]. Moreover, exendin-4 increases PI3K and AMPK levels, promotes autophagy, and protects against apoptotic cell death [77]. Likewise, semaglutide stimulates the PI3K/AKT pathway and increases glucose transporter GLUT4 in the hippocampus from HFD-fed mice [75]. Pioglitazone enhances brain insulin sensitivity in HFD-fed mice by activating the AKT/GSK3ß pathway, without affecting CSF insulin levels [138]. Conversely, rosiglitazone shows mixed effects, partially downregulating insulin signaling proteins (IR, IRS1 and AKT) in T2D [143], but enhancing insulin signaling ex vivo in PreDM rats [141].

Metformin reverses brain insulin resistance by promoting AKT and IRS1 phosphorylation [187, 191, 194, 196, 197], though one study did not observe these effects [198]. On the other hand, results on AMPK and mTOR activation are largely inconsistent [192, 194, 197]. Additionally, metformin induces autophagy, decreases JNK activation, increases *Nrf2* expression and promotes atypical PKC ζ/λ phosphorylation [188, 190, 194, 197]. The SU glimepiride improves brain insulin signaling by enhancing AKT and IRS1 phosphorylation and inhibiting GSK3ß activation [187]. The SGLT2i empagliflozin and DPP4i sitagliptin restore insulin sensitivity in HFD/STZ mice by decreasing IRS1 phosphorylation at Tyr632, associated with insulin resistance, and boosting AKT phosphorylation [208]. Conversely, sitagliptin enhances GSK3ß activity in Otsuka Long Evans Tokushima Fatty rats [224] And upregulates adiponectin receptor 1 gene expression in the hypothalamus [227]. Alogliptin and vildagliptin restore AKT and ERK activation [225, 226], with alogliptin also increasing CREB phosphorylation [226], whereas vildagliptin inactivates the STAT3/JAK pathway and reduces hippocampal apoptosis [225]. Additionally, intranasal insulin increases CSF insulin levels and restores brain insulin signaling [66], unlike subcutaneous insulin [196, 277], which has limited effects on the AKT/GSK3ß pathway [67].

##### Effect of antidiabetic drugs on cognitive performance

Exendin-4 improves cognitive performance in genetic and diet-induced T2D models across several tests, including NOR, MWM and the Barnes maze [66, 76], whereas liraglutide enhances spatial learning and memory in the MWM and NOR in db/db and PreDM mice [74, 79]. Similarly, semaglutide treatment in HFD-fed mice improves performance in elevated plus maze and T-maze tests [75], and Val8GLP1 administration enhances NOR performance, producing better effects when combined with metformin [73].

TZDs pioglitazone and rosiglitazone improve cognitive function in multiple tests, including MWM, NOR, Y-maze and attentional set shifting [137, 139, 140, 142–144]. Nevertheless, metformin shows mixed results, with some studies noting improvements in MWM, Y-maze and T-maze tests [188, 191, 194, 195, 199], while others found no benefits in the Barnes maze, operant-based task or fear conditioning tests [190, 198, 200].

The SU glibenclamide enhances performance in Y-maze and MWM tests in T2D rats [232]. The SGL2i empagliflozin improves spatial learning and memory in the MWM test and NOR [208, 210, 211] and luseogliflozin reverses cognitive impairments in the NOR and the eight-arm water maze [209]. DPP4i sitagliptin and vildagliptin improve cognition in NOR, MWM and the hole board test [208, 225, 227]. Intranasal insulin enhances performance in NOR and MWM, while subcutaneous insulin shows no effect [66]. Finally, the dual amylin/calcitonin receptor agonist KBP-36 improves MWM performance in Zucker diabetic fatty rats [80].

### Preclinical models of AD concomitant with metabolic impairment

**Fig. 5:**
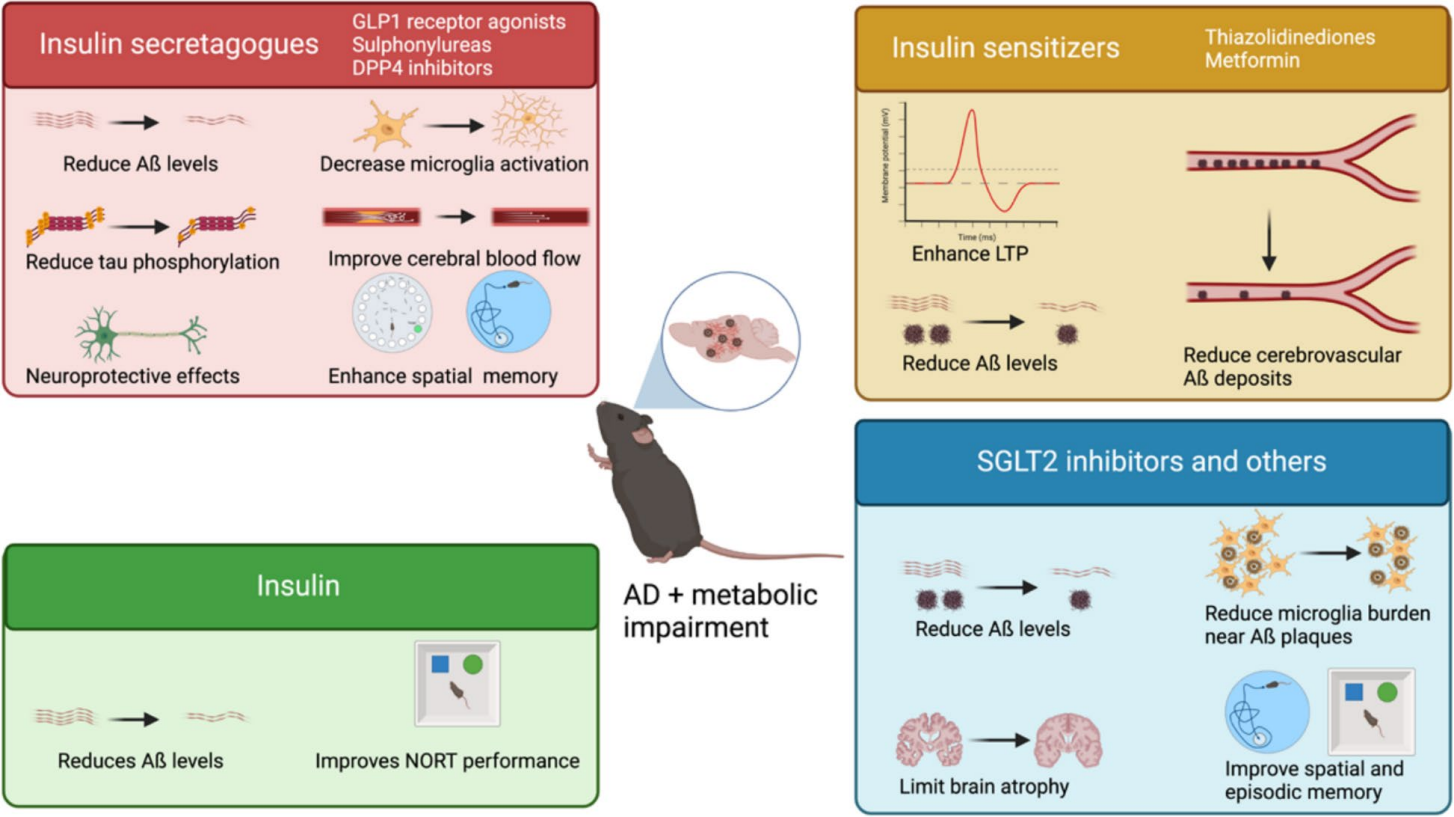
Beneficial effects of antidiabetic drugs as disease-modifying therapies for dementia on preclinical models of AD concomitant with metabolic impairment. Created with BioRender.com

Age is the main risk factor for AD, but metabolic conditions like T1D, T2D, and PreDM also increase AD risk and cognitive decline. Murine models are commonly used to study how diabetes-related dysregulation interacts with AD-related neurodegeneration, mirroring clinical observations. Key findings are summarized in Fig. 5, with references in Table 1.

#### Effect of antidiabetic drugs on AD neuropathological hallmarks and markers

GLP1 analog exendin-4 has lowers soluble Aß levels in the hippocampus and cortex, reduces tau hyperphosphorylation and increases neuron numbers in an AD-T1D mouse model [81, 82]. In contrast, exenatide has limited impact on Aß deposition or tau hyperphosphorylation in AD-PreDM mice, but promotes neuroprotection by increasing BDNF and PSD95 levels [78]. On the other hand, liraglutide reduces oligomeric Aß, insoluble Aß40 and Aß42, tau hyperphosphorylation and brain atrophy, preserving neuronal health in AD-T2D mice [79]. On the contrary, in an *ApoE* mouse model fed with HFD, the TZD pioglitazone shows no effect on Aß levels in wildtype or *ApoE* ε3/4 genotypes but reduces Aß40 in ApoE *knockout* mice [145]. Notably, pioglitazone reduces tau phosphorylation in insulin-resistant *ApoE* ε3 mice but exacerbates it in *ApoE* ε4 littermates, indicating greater responsiveness of the *ApoE* ε3 allele to TZD treatment [145].

Metformin protects against synaptic plasticity impairment by enhancing LTP in AD-PreDM mice [201], while reducing hippocampal Aß levels (soluble and insoluble Aß42, insoluble Aß40 and Aß plaque area), and increasing IDE expression in AD-T2D mice [202]. Similarly, empagliflozin reduces amyloid plaque burden, soluble Aß40 and brain atrophy, and increases cortical thickness and neuronal density in AD-T2D mice [211]. In this line, DPP4i vildagliptin reduces hippocampal Aß42 levels and preserves neuronal integrity in AD-PreDM mice [225], while also decreasing APP expression and tau hyperphosphorylation in an AD-T2D rat model [228]. Additionally, acute intravenous insulin administration decreases soluble Aß40 and Aß42 levels, reduces the soluble/insoluble Aß42 ratio, decreases BACE1 expression and increases X11α in HFD-fed 3xTg-AD mice [279]. Conversely, α-glucosidase inhibitor acarbose does not seem To reduce cerebral amyloid pathology neither tau phosphorylation in 3xTg-AD mice fed high-fat/high-sugar diet, though it shows a trend to reduce total tau protein [288].

#### Effects of antidiabetic drugs on oxidative stress, neuroinflammation, vascular damage and brain glucose metabolism

GLP1 analogs have also shown beneficial effects in neuroinflammation. Exenatide reverses HFD-induced increases in NFκB, PPAR-α and PPAR-γ in the cortex, hippocampus And cerebellum of 3xTg-AD mice [78]. Liraglutide reduces microglia burden in the cortex, and empagliflozin lowers microglia accumulation near amyloid plaques, while both drugs also reduce spontaneous hemorrhage in the cortex from AD-T2D mice [79, 211]. Additionally, metformin protects brain vasculature by reducing cerebrovascular Aß deposits in AD-T2D mice [202]. DPP4i vildagliptin reduces hippocampal FOXO1 and TNF-α levels in AD-PreDM mice [225] and decreases MDA levels, preventing lipid peroxidation, in AD-T2D mice [228]. Moreover, linagliptin restores cerebral blood flow in AD-PreDM mice [229]. On the contrary, acarbose shows sex-specific effects in 3xTg-AD mice fed high-fat/high-sugar diet: it reduces Iba1 levels in the hippocampus only in female mice, whereas does not modulate GFAP expression [288].

#### Effect of antidiabetic drugs on brain signaling pathways and cognitive performance

Exendin-4 improves learning in the Barnes maze test in AD-T1D mice [81]. Exenatide in AD-PreDM mice increases IRS1 phosphorylation and restores the BDNF/TrkB axis, while decreasing proBDNF and P75 neurotrophin receptor levels, without affecting MWM performance [78]. In contrast, liraglutide and empagliflozin enhance spatial and episodic memory in AD-T2D mice in the MWM and NOR tests [79, 211]. On the other hand, DPP4i vildagliptin in AD-PreDM rats shows antiapoptotic effects, increases klotho expression, activates AKT and inactivates STAT3/JAK2 pathways [225]. Vildagliptin improves spatial learning and memory in the MWM in AD-PreDM rats [225], and enhances performance in the forced swimming test and passive avoidance test in AD-T2D rats [228], while linagliptin restores spatial reference memory in P301S mice fed HFD [229]. Remarkably, a single intraperitoneal insulin injection improves NOR performance in 3xTg-AD mice fed HFD [279], and similarly intranasal insulin enhances NOR in both *ApoE ε3* and *ApoE ε4* mice fed HFD, whereas it only improves MWM performance in *ApoE ε3* mice [278]. The α-glucosidase inhibitor acarbose increases AKT phosphorylation specifically in female 3xTg-AD mice fed high-fat/high-sugar diet, indicating increased mTOR activity, and it shows sex-specific effects on autophagy: it decreases p62 levels in Male 3xTg-AD mice, suggesting enhanced autophagic flux, whereas elevates beclin-1 levels in females, suggesting autophagic initiation [288]. Finally, acarbose enhances learning and memory processes in the Barnes Maze test specifically in 3xTg-AD female mice fed high-fat/high-sugar diet [288].

### Preclinical studies closing remarks

Preclinical studies suggest antidiabetic drugs have neuroprotective potential in AD and related dementias. GLP1 analogs, TZDs, SGLT2 inhibitors, DPP4 inhibitors, SU and insulin show overall benefits for amyloid and tau pathologies, neuron health, inflammatory and vascular alterations, alongside improved cognition in animal models mimicking AD and/or diabetes. Altogether, these preclinical findings show promising results and suggest that repurposing antidiabetic drugs could pave the way for new therapeutic strategies in Alzheimer's treatment.

### Clinical studies in AD and AD-like dementia patients

Given the large number of studies on the effects of antidiabetic treatments in the brain —and the considerable diversity in methodologies— clinical trials and observational studies (both longitudinal and cross-sectional) have been analyzed separately (Supplementary Table 3). This structure aims to bring clarity and coherence to the available evidence.

#### Effect of antidiabetic drugs on AD neuropathological hallmarks and markers

##### Clinical trials in AD and AD-like dementia patients

Previous studies have reported differences in [^11^C]PIB PET to measure Aβ load, in some brain regions from AD patients without diabetes treated with liraglutide [299]. A pilot study, in non-diabetic individuals, with other GLP1 analog, exenatide, revealed no differences in CSF biomarkers (Aβ42, total tau and p181 tau) or plasma Aβ40 and Aβ42 levels. However, exenatide might reduce Aβ42 in plasma neuronal extracellular vesicles, as a relevant target for biomarker responses in AD [300]. While this study did not show differences in cortical thickness and volume by MRI, another short-term trial with cognitively normal, late middle-aged individuals on liraglutide, with subjective cognitive complaints, revealed an increase in connectivity with the bilateral hippocampus, suggesting that GLP1 analogs might be useful at this level [301].

The effects of TZD in AD markers appear limited. In a six-month trial involving AD patients with T2D, pioglitazone affected plasma Aβ40 or 42 levels, whereas the Aβ40/Aβ42 remained stable in the treatment group but increased in the control group [302]. Similarly, rosiglitazone administration to patients with AD or MCI did not affect plasma Aβ levels [303] nor did it affect brain atrophy [304].

Biguanides have been more extensively studied and a randomized placebo-controlled crossover study with metformin in non-diabetes patients with MCI due to AD, revealed no significant changes in CSF levels of Aβ42, Total tau, or phospho-tau after 8 weeks of treatment, although these results were based on pilot data [305]. Similarly, in another pilot randomized placebo-controlled trial involving patients with amnestic MCI, plasma Aβ42 increased in the metformin group and decreased in the placebo group, although none of the changes were statistically significant [306]. It is important to note that both studies were of short duration—8 weeks And 4 weeks, respectively. In contrast, a more recent 20-week pilot clinical trial in non-diabetic patients with MCI related to AD reported metformin-induced alterations in plasma and CSF proteins associated with inflammation (AZU1, CCL11, IL-32), apoptosis (CASP-3), and neuroprotection (PRTN3), supporting potential neuroprotective effects of metformin beyond its established antidiabetic action [307].

A small trial with AD patients receiving intravenous insulin revealed that APOE ε4 non-carriers Had lower APP levels when insulin levels reached 85 μU/ml, whereas individuals carrying one APOE ε4 allele showed increased APP levels [308]. Similarly, intravenous insulin infusion to healthy older participants led to an increase of CSF Aβ42 levels, which related with memory residual values, supporting an overall interaction between insulin, Aβ42 and cognition [309]. In contrast, a pilot study administering 20 or 40 IU of intranasal insulin to patients with MCI reported no significant changes in CSF levels of Aβ40, Aβ42 and tau [310]. A subsequent larger study with AD patients confirmed these findings, showing no significant effects on CSF biomarkers including Aβ40, Aβ42, Aβ42/Aβ40, total tau, p181tau And Aβ 42/total tau ratios [311]. On the other Hand, plasma biomarker results Have been more variable. In one small trial involving AD or MCI patients treated with 20 IU of intranasal insulin, fasting plasma Aβ40 levels and Aβ42/Aβ40 ratio increased compared to placebo, while postprandial analysis showed no changes in Aβ40, reduced Aβ42 levels, and increased Aβ40/42 ratio in the insulin-treated groups [312]. Also, another small study with cognitively impaired participants found no dose–response effect for plasma Aβ40 levels. However, Aβ42 levels increased in APOE ε4 carriers receiving 10 IU of insulin, and a U-shaped dose–response pattern (up to 60 IU) was observed for Aβ42 levels in APOE ε4 non-carriers [313].

Regarding neuroimaging Outcomes, treatment with intranasal insulin for 12 months did not significantly alter temporal-parietal cortical thickness. However, significant changes were noted in white matter hyperintensities, including global, deep white matter, corpus callosum, and lobar regions (frontal, temporal, parietal, and occipital) [314]. Also, white matter hyperintensity volume increase correlated with Aβ42 CSF levels, and corpus callosum and deep white Matter hyperintensity volumes correlated with reductions in Aβ 42/tau ratio, when both placebo and treated groups were analyzed together. In the insulin-treated group alone, parietal white matter hyperintensity volumes inversely correlated with CSF Aβ40 levels [314]. Bibliographic references are compiled in Table 3.

**Table 3:** Summary of clinical studies assessing the effects of antidiabetic drug groups across different populations. Cell values indicate the number of studies as well as the specific references on a drug group within a specific population

	**MCI, AD and AD-like dementia**	**Adult population and/or at risk of AD**	**Metabolic impairment**	**Cognitive and metabolic impairment**
**GLP1 receptor agonists**	6 [299–301, 315–317]	-	11 [318–328]	-
**TZD/PPAR-γ agonists**	10 [303, 304, 317, 329–335]	4 [336–339]	11 [134, 318, 320, 325, 326, 340–345]	7 [135, 302, 346–350]
**Biguanides**	4 [306, 307, 317, 335]	7 [305, 337, 338, 351–354]	28 [318, 320, 325, 340, 355–379]	9 [346, 347, 349, 350, 380–384]
**SGLT2i**	1 [317]	-	12 [318, 320, 322, 325, 327, 328, 385–390]	1 [135]
**DPP4i**	1 [317]	1 [391]	15 [318, 320, 322, 324–326, 328, 386–388, 392–396]	4 [135, 349, 381, 397]
**Sulphonylureas**	2 [317, 335]	3 [337, 338, 352]	17 [318, 320, 322, 324, 325, 340, 355, 357, 359, 369, 373–376, 379, 389, 392]	4 [135, 346, 349, 350]
**Insulin**	18 [308, 310–314, 317, 398–408]	6 [309, 337, 352, 409–411]	13 [318, 320, 323, 325, 340, 355, 357, 359, 392, 412–415]	7 [347, 349, 350, 416–419]
**Others**	1 [408]	3 [410, 411, 420]	7 [320, 340, 345, 377, 414, 415, 421]	5 [135, 416–419]
**Combined treatments**	-	-	4 [342, 360, 369, 422]	4 [346, 382, 416, 419]

##### Observational studies in AD and AD-like dementia patients

Observational studies have not addressed the effects of some antidiabetic groups on AD pathology, including GLP1 analogs or TZD. On the other hand, a small study analyzing plasma Aβ42 levels revealed no significant differences in patients taking biguanides [355], while a larger study with diabetic patients treated with metformin during early stages of AD showed higher levels of Aβ and lower levels of tau and phospho-tau in CSF [380]. While postmortem AD pathology (amyloid burden and tau density) does not seem To be affected in older adults treated with metformin for An average of 8 years when compared with non-users, as revealed in a cross-sectional study [351], neuroimaging findings in patients with MCI and diabetes are more mixed. One study reported that metformin users had larger cortical thickness and hippocampal volumes after adjusting for APOE ε4 genotype [380]. In contrast, another study found no significant effects of metformin administration for at least two years to MCI diabetic patients on the hippocampus, occipital and temporal lobes, although treated patients had higher right and left parietal lobes volumes as well as a larger left cingulate and frontal lobe [356]. Nevertheless, two large cross-sectional studies with diabetes patients found no differences in white matter hyperintensity volumes, total brain volumes, or specific structures including the hippocampus, parahippocampus and precuneus between metformin users and non-users [357, 358].

When SGLT2i are assessed in T2D patients, these are less likely to have impaired CSF p-tau levels and altered t-tau/Aβ_42_ ratio, although the effects seem to be dependent on metabolic control [318]. Other studies have reported that diabetes patients on DPP4i Have lower global And regional amyloid burdens, assessed by 18F-florbetaben amyloid positron emission tomography (PET) [397]. On the contrary, no significant differences have been observed in plasma Aβ42 levels after SU treatment, as reported in both longitudinal and cross-sectional studies [352, 355]. Some longitudinal studies have also analyzed the effect of insulin on AD-related pathology. A small study revealed higher plasma Aβ42 levels in insulin users [355], although it has also been reported that insulin use reduces the odds of pathological CSF Aβ42 levels in T2D patients after adjusting for educational level and cardiovascular comorbidities, and higher insulin doses also reduce the odds of t-tau/Aβ42 alterations, while detected effects seem to be dependent on metabolic control [318]. Nevertheless, other studies have shown higher medial temporal lobe atrophy when insulin users are compared with non-users [355]. At this point, it is worth noting that many of these studies have analyzed different factors and subgroups, which makes it somewhat difficult to interpret the actual findings or outcomes. Importantly, patients on insulin tend to have more severe diabetes, making this population more susceptible to other complications.

Studies on postmortem tissue from patients treated with antidiabetic treatments are limited and have mostly compared the effects of different hypoglycemic agents. In this sense, a study in T2D patients with no dementia, that included race as a covariable, revealed no effects of insulin on traditional measures of AD-neuropathology, although lower Aβ42 levels were detected when compared with biguanide or SU users [359]. Another small study compared insulin alone, SU, metformin and TZD, or a combination of insulin + SU or insulin + metformin, and revealed alterations in mRNA transcripts in the brain related to insulin signaling, vascular markers, inflammation and neuron integrity. Although sample sizes were small and individual antidiabetic agents—and their combinations—were analyzed collectively, findings suggest meaningful molecular effects. For instance, treatment appeared to restore expression of SNAP25 and NEUROD2 in both whole parahippocampal gyrus and endothelial-cell–enriched isolates [346]. Also, a postmortem study of AD patients without prior antidiabetic treatment found that those receiving combined insulin plus another antidiabetic agent had significantly lower amyloid plaque density in the hippocampus, entorhinal cortex, amygdala, and frontal cortex—while neurofibrillary tangle burden remained unaffected [416]. Bibliographic references are compiled in Table 3.

#### Effect of antidiabetic drugs on neuroinflammation, vasculature and glucose metabolism

##### Clinical trials in AD and AD-like dementia patients

Many antidiabetic treatments also Have Anti-inflammatory properties And a small clinical trial involving patients with probably AD demonstrated that treatment with pioglitazone for 6 months resulted in significant changes in TNF-α levels, which were significantly correlated with cognitive performance [329]. Surprisingly, 40 IU of intranasal insulin for 12 months led to increased levels of IFN-γ and eotaxin, along with decreased IL-6 levels in CSF. Furthermore, changes in inflammatory and immune markers found to correlate with pathological markers of AD (Aβ40, 42, tau, phospho-tau and different ratios) [398]. However, the interpretation of these findings remains challenging due to the small sample size and the variability of effects depending on the specific marker and study group.

In a 26-week trial, liraglutide treatment was associated with an increase in BBB glucose transfer capacity, as assessed by F18 fluorodeoxyglucose PET imaging [315]. Interestingly, clearance of [^11^C]PIB, as surrogate measure of cerebral blood flow, was increased in the placebo group but not in the liraglutide group. In a related study, brain glucose metabolism was reduced in the placebo group, and basal-to-6-months glucose metabolism ratio was reduced in the cingulate and occipital lobes compared with the liraglutide-treated group [299], whereas a study using functional near‐infrared spectroscopy showed that liraglutide treatment led to an overall increment in oxyhemoglobin concentration in the dorsolateral prefrontal and orbitofrontal cortices, suggesting enhanced cortical perfusion or activity [319].

On the other hand, TZD treatments have shown mixed results. Pioglitazone administered over six months improved regional cerebral blood flow in the bilateral parietal lobes and parts of the frontal lobe in AD patients [302]. Additionally, a pilot study reported that metformin treatment for 8 weeks led to significantly increased blood flow in the superior and middle orbitofrontal cortices in patients with MCI or AD [305]. Similarly, intranasal insulin (20 or 40 IU) reduces the progression of cerebral hypometabolism in various brain regions [310]. Likewise, vascular Markers assessed in 12-month pilot study with AD patients treated with intranasal insulin showed that intercellular adhesion molecule-1, vascular cell adhesion molecule-1, basic fibroblast growth factor or vascular endothelial growth factor, among others, were differently correlated with other AD markers (Aβ or tau), when compared with the control group [398].

##### Observational studies in AD and AD-like dementia patients

Molecular analyses of the parahippocampal gyrus and endothelial cell-enriched isolates from AD patients, both with and without comorbid diabetes mellitus, have revealed that antidiabetic treatments May normalize the expression of key vascular And metabolic Markers, such as mRNA levels of neural cell adhesion molecule 1 (NCAM1) and aquaporin-4. Additionally, insulin-like growth factor 1 receptor (IGF1R) expression was significantly reduced in treated patients, alongside decreased expression of glucose transporter GLUT4 (SLC2A4), AKT1, and AKT3, in both endothelial and non-endothelial cell preparations [346]. These findings suggest a potential normalization of glucose transport and insulin signaling pathways in response to antidiabetic medication. However, another study evaluating different insulin-stimulating agents, including SU, repaglinide, GLP1 analogs, DPP4i, GLP1, metformin and pioglitazone reported no significant effects on CSF markers of AD (Aβ42, total tau, p-tau, total tau/Aβ42) in patients with T2D [318]. This highlights the complexity of translating peripheral metabolic interventions into central biomarker changes in AD.

#### Effect of antidiabetic drugs on cognitive performance

**Fig. 6:**
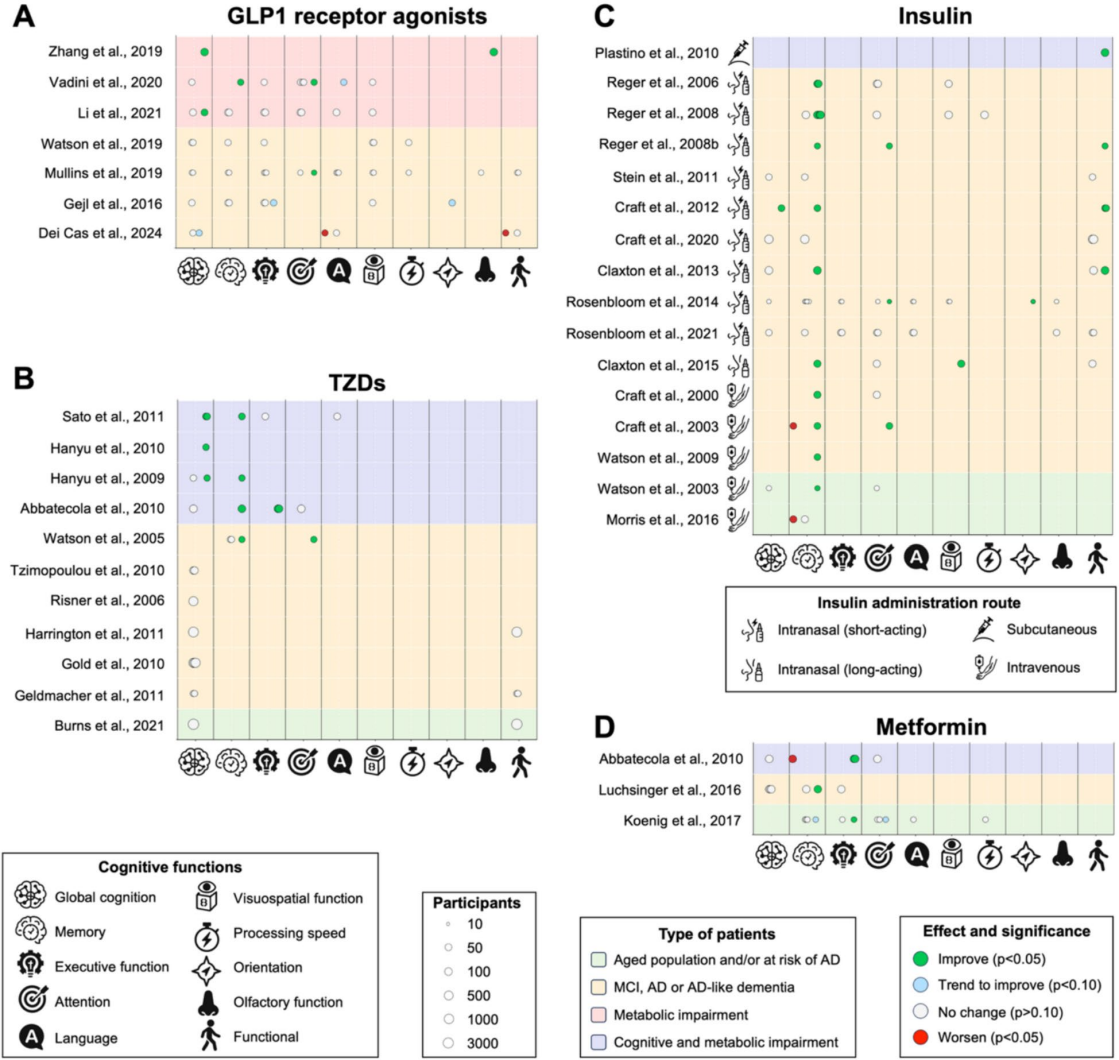
Cognitive effects of antidiabetic treatments in interventional clinical studies. Summary of published clinical trials evaluating the cognitive impact of four classes of anti-diabetic drugs: (**A**) GLP-1 receptor agonists, (**B**) thiazolidinediones (TZDs), (**C**) insulin, and (**D**) metformin. Each row represents a study, with cognitive domains assessed indicated by icons. Circle colors denote the direction and statistical significance of the effect on cognition, while circle size corresponds to the number of participants per study. Background shading indicates patient type. Cognitive domains were evaluated using standard neuropsychological tests, such as Alzheimer’s Disease Assessment Scale (ADAS)-cognitive subscale (ADAS-cog), Alzheimer’s Disease Cooperative Study Clinical Global Impression of Change for Mild Cognitive Impairment (ACDS CGIC-MCI), Clinician’s Interview-Based Impression of Change Plus Caregiver Input (CIBIC +), Clinical Dementia Rating Score (CDR), Dementia Severity Rating Scale (DSRS), Mini-Mental State Examination (MMSE), Montreal Cognitive Assessment (MoCA), Nurses’ Observation Scale for Geriatric Patients, Wechsler Abbreviated Scale of Intelligence (WASI) and Wechsler Adult Intelligence Scale for global cognition; Buschke Delayed Memory Test, Buschke Selective Reminding Test (SRT), California Verbal Learning Task (CVLT), Delayed matching to sample, Dot counting N-back, Hopkins Verbal Learning Test, Incident Recall, Logical Memory II Delayed Paragraph Recall, Memory and Executive Screening (MES), Memory Interference List Recall, Paired Associates Learning, Repeatable Battery for the Assessment of Neuropsychological Status (RBANS), Rey Auditory-Verbal Learning Test (RAVLT), Story Recall (immediate and delayed), Verbal Reproduction, Wechsler Memory Scale Logical Memory Subtest, and Word List Learning/Recall for memory function; Delis-Kaplan Executive Functioning System (DKEFS), Difference Between Trail Making Test Part B and Part A (DIFFBA), Digit Span Backwards, Frontal Assessment Battery (FAB), Inhibition, MES Executive, Mental Control, Time Stimation and Trail Making Test B (TMT-B) for executive function; Delayed Matching to Sample, Digit Span Forward, Stroop Color-Word Interference Task, Trail Making Test A (TMT-A) and Visual Searching Matrices (VSM) for attention; American National adult reading test (ANART), Animal Naming Test, Boston Naming Test, Category Fluency, Controlled Oral Word Association Test (COWAT), Phonemic Verbal Fluency Test, RBANS Semantic Fluency and Semantic Verbal Fluency Test for language function; Benton Visual Retention Task (BVRT), Rey Complex Figure Test (RCFT), Rey-Osterrieth complex figure test (ROCF) and Self-ordered pointing task (SOPT) for visuospatial function; Digit Symbol, Purdue Pegboard, Reaction Time and Wechsler Adult Intelligence Scale Digit-symbol for processing speed; RBANS Orientation for orientation function; Alberta Smell Test, Olfactory Function, Sniff Magnitude Test (SMT) and University of Pennsylvania Smell Identification Test (UPSIT) for sensory function; Alzheimer's Disease Cooperative Study-Activities of Daily Living (ADCS-ADL), ADCS-ADL scale for MCI (ADL-MCI), Alzheimer's Disease Functional Assessment and Change Score, Clinician's Global impression (CGI), Dementia severity rating scale (DSRS), Disability Assessment in Dementia, Functional Assessment Questionnaire (FAQ) and Instrumental Activities of Daily Living (IADL) for functional assessment

The effects of antidiabetic treatments on cognition and the risk to develop dementia, or AD specifically, are the most common studies in healthy subjects, patients with T2D and those with cognitive impairment or AD. Since most studies do not report the raw data necessary for meta-analysis, we have synthesized the available cognitive outcomes qualitatively (Fig. 6). Bibliographic references are compiled in Table 3. A summary of the cognitive outcomes in clinical trials and observational studies is provided in Table 4.

**Table 4:** Summary of clinical studies and cognitive outcomes for individual or combined therapies as well as compared treatments. Cognitive outcomes are represented with symbols:  improvement: cognitive improvement or reduced risk of dementia (green),  no effect (grey),  worsening: cognitive decline or increased risk of dementia (red),  mixed results (orange)

Study type	Antidiabetic group	Treatment	Patient group	Cognitive outcomes/risk of dementia
**Clinical trial**	**GLP1 analogs**	Liraglutide	Subjective cognitive complaints	No effect [301]
			Nondiabetic AD-patients	No effect [299]
			Metabolic impairment	Improvement [319]
		Exenatide	Metabolic impairment	Improvement [360]
			MCI	No effect [316]
	**TZD**	Pioglitazone	MCI	No effect [336]
		Rosiglitazone	Aged population	No effect [304]
			AD/Cognitive impairment	Mixed results ( [272–274]; APOE ε4 carriers [333]; APOE ε4 non-carriers [333])
	**Biguanides**	Metformin	Cognitive impairment	Improvement [305] Mixed results [382]
			Cognitive and metabolic impairment	Mixed results [306]
	**Insulin** **(intravenous)**		MCI/AD	Mixed results ( APOE ε4 homozygote carriers [308]; APOE ε4 non-carriers [399–401]; APOE ε4 carriers [399]; [409])
	**Insulin** **(intranasal)**		MCI/AD	Mixed results ( [310, 313, 405]; APOE ε4 non-carriers [402]; APOE ε4 carriers [402]; [407]; [311])
		Glulisine	MCI/AD	Mixed results ( [403]; [404])
		Detemir	MCI/AD	Mixed results ( overall, APOE ε4 carriers, APOE ε4 non-carriers [406])
	**Compared treatments**	Oral antidiabetics vs. oral antidiabetic+ insulin	Cognitive and metabolic impairment	Improvement(with oral antidiabetics+insulin) [419]
		Metformin vs. metformin+liraglutide	Metabolic impairment	Improvement(with metformin+liraglutide) [321]
		Metformin vs. SU	MCI, AD and AD-like dementia	Improvement (with metformin) [300, 301]
		Metformin vs. metformin+rosiglitazone	Cognitive and metabolic impairment	Improvement (with metformin+rosiglitazone) [382]
**Observational (cross sectional)**	**TZD**		Metabolic impairment	Worsening [392]
	**Biguanides**		Cognitive and metabolic impairment	Mixed results ( [364]; [349])
		Metformin	Metabolic impairment	Worsening [392]
			Cognitive and metabolic impairment	Improvement [380]
	**DPP4i**		Cognitive and metabolic impairment	Improvement [349]
	**SU**		Cognitive and metabolic impairment	No effect [349]
	**Insulin**		Metabolic compromise	Mixed results ( [392, 417]; [414])
			Cognitive and metabolic impairment	No effect [349]
	**Other antidiabetic drugs and combined treatments**	Hypoglycemic agents	Metabolic impairment	Worsening [417]
		FPP4i+SU or DPP4i+insulin	Metabolic impairment	Worsening [392]
		α-glucosidase inhibitors, TZD+insulin or TZD+insulin+SU	Metabolic impairment	No effect [392]
	**Compared treatments**	GLP1 analogs vs. SU or DPP-4 inhibitors	Metabolic impairment	Improvement (with GLP1 analogs) [324]
		Semaglutide vs. other GLP1 analogs, insulin, metformin, DPP4i, SGLT2i, SU or TZD	Metabolic impairment	Improvement (with semaglutide) [323]
		Metformin vs. other antidiabetic drugs	Metabolic impairment	Improvement (with metformin) [354, 411]
		Metformin vs. SU (glyburide and glipizide)	Metabolic impairment	Improvement (with metformin) [326]
		Metformin vs. GLP1 analogs (exenatide, liraglutide, dulaglutide)	Metabolic impairment	Improvement (with GLP1 analogs) [326]
		Metformin vs. nateglinide	Metabolic impairment	Improvement (with metformin) [326]
		Metformin vs. rosiglitazone	Metabolic impairment	Improvement (with rosiglitazone) [326]
		Metformin vs. metformin+insulin	Aged population and/or at risk of AD	Improvement (with metformin) [411]
		Dapagliflozin and empagliflozin vs. dulaglutide	Metabolic impairment	Improvement (with dapagliflozin and empagliflozin) [327]
		DPP4i vs. insulin or SU	Cognitive and metabolic impairment	Improvement (with DPP4i) [349].
**Observational (longitudinal)**	**GLP1 analogs**		Cognitive and metabolic impairment	No effect [347]
			Metabolic impairment	Improvement [320]
			AD	Improvement [317]
	**TZD**		Metabolic impairment	No effect [233, 276]
			Cognitive and metabolic impairment	Worsening [381]
			Aged population	Worsening [338]
			AD	Improvement [317]
			Metabolic impairment	Improvement [134, 320, 343, 347]
		Pioglitazone	Metabolic impairment	Mixed results ( [342]; [339, 344])
			Cognitive impairment	Improvement [334]
	**Biguanides**		Aged population	Worsening [352]
			Metabolic impairment	Improvement [320]
			Cognitive and metabolic impairment	Mixed results ( [347]; [349])
		Metformin	Aged population	Mixed results ( [351, 363]; [353, 363, 364])
			Metabolic impairment	Mixed results ( [358, 366, 369–372, 384]; [361]; [337, 340, 353, 357, 362, 365])
			Cognitive and metabolic impairment	Mixed results ( [135, 382, 383]; in APOE ε4 non‐carriers but in APOE ε4 carriers [381])
	**SGLT2i**		Metabolic impairment	Improvement [385]
			Cognitive and metabolic impairment	No effect [347]
	**DPP4i**		Metabolic impairment	Improvement [320]
			Cognitive and metabolic impairment	Mixed results ( [347]; [349, 381, 397])
		vildagliptin	Metabolic impairment	Mixed results ( [394]; [395])
		sitagliptin; linagliptin; saxagliptin; alogliptin	Metabolic impairment	Improvement [395]
	**SU**		Metabolic impairment	Mixed results ( [320, 337, 357]; [317, 340, 369])
			Metabolic and cognitive impairment	No effect [347] [349, 381]
	**Insulin**		Aged population	No effect [352]
			Metabolic compromise	Mixed results ( [320, 337]; [350, 408]; [317, 340, 342, 357, 410, 412, 413])
			Metabolic and cognitive impairment	Worsening [347, 418]
	**Other antidiabetic drugs and combined therapies**	Hypoglycemic agents	Aged population and/or at risk of AD	Worsening [410, 418, 420]
		α-glucosidase, non-SU insulin secretagogues or non-SU insulin secretagogue combination therapy	Metabolic impairment	No effect [340]
		Insulin, metformin, SU, TZD, DPP4i, GLP1 analogs, SGLT2i combinations	Metabolic impairment	Improvement (with all combinations but SU) [320]
		metformin+TZD, metformin+SU+meglitinide, metformin+SU+glucosidase inhibitor, metformin+SU+TZD, metformin+meglitinide+TZD, metformin+glucosidase inhibitor+TZD	Metabolic impairment	Worsening [421]
		Biguanides or biguanide+other antidiabetic drug	Metabolic impairment	Improvement [421]
	**Compared treatments**	Semaglutide vs. sitagliptin or glipizide	Aged population and/or at risk of AD	Improvement (with semaglutide) [322]
		Semaglutide vs. metformin	Middle aged adults	No effect [322]
		Metformin vs. other oral antidiabetics	Metabolic impairment	Improvement (with metformin) [376–379, 411]
		Metformin vs. SU	Metabolic impairment	Improvement (with metformin) [338, 373–376, 379].
			Aged population	Improvement (with metformin) [338]
		Metformin vs. TZD	Aged population	Improvement (with metformin) [338]
		Metformin vs. metformin+other oral antidiabetics	Metabolic impairment	No effect [325]
		Metformin vs. metformin+insulin	Metabolic impairment	Mixed results ( (with metformin+insulin)[396]; [325])
		Metformin vs. metformin+sitagliptin	Metabolic impairment	Improvement (with metformin+sitagliptin) [396]
		Metformin vs. metformin+vildagliptin	Metabolic impairment	Improvement (with metformin+vildagliptin) [391]
		Metformin vs. metformin+SU	Metabolic impairment	Improvement (with metformin+SU) [369]
		Metformin+pioglitazone vs. metformin+rosiglitazone, metformin+SU, metformin+insulin, metformin+meglitinide or metformin+acarbose	Metabolic impairment	Improvement (with metformin+pioglitazone) [422]
		Metformin vs. metformin+SU+DPP4i or metformin+glucosidase inhibitor+DPP4i	Metabolic impairment	Improvement (with metformin+SU+DPP4i and metformin+glucosidase inhibitor+DPP4i) [421]
		SU vs. SU+other antidiabetic drugs	Metabolic impairment	Improvement (with SU+other antidiabetics) [421]
		TZD vs. SU	Aged population	No effect [338]
		TZD vs. α-glucosidase inhibitors	Metabolic impairment	Improvement (with TZD) [345]
		SGLT2i vs. DPP4i	Metabolic impairment	Improvement (with SGLT2i) [386–388]
		SGLT2i vs. SU	Metabolic impairment	Improvement (with SGLT2i) [389]
		DPP4i vs. dapagliflozin or empagliflozin	Metabolic impairment	Improvement (with dapagliflozin and empagliflozin) [387]
		DPP4i vs. canagliflozin	Metabolic impairment	No effect [387]
		SGLT2i+metformin vs. GLP1 analogs+metformin	Metabolic impairment	Improvement (with SGLT2i+metformin) [328]
		SGLT2i and SGLT2i+metformin, SU, meglitinide, DPP4i, TZD, α-glucosidase inhibitor or insulin vs. oral antidiabetic drug (other than SGLT2i) with or without insulin	Metabolic impairment	Improvement (with SGLT2i or SGLT2i+metformin, SU, meglitinide, DPP4i, TZD, α-glucosidase inhibitor or insulin) [390]

##### **GLP1 analogs**

**Clinical trials in AD and AD-like dementia patients**

Clinical studies investigating the cognitive effects of GLP1 receptor agonists Have produced mixed results. A short-term study involving participants with subjective cognitive complaints found no significant effects in a comprehensive battery of cognitive tests after 12 weeks on liraglutide [301] and Similar Outcomes have been reported on non-diabetic AD patients treated with Liraglutide for 6 months [299]. The limited repercussion of GLP1 analogs in cognition might may reflect a delay between neurobiological changes, like better intrinsic connectivity, and observable improvements in cognition [301]. In contrast, a study with T2D reported overall improvements in cognitive performance following 12 weeks of liraglutide treatment [319]. Furthermore, a targeted study involving obese individuals with poor glycemic control and metformin monotherapy, showed beneficial effects of exenatide on cognition and olfactory function, as possible early predictors of neurodegeneration. These effects were accompanied by increased activation in the right parahippocampal region, as measured by MRI [360]. However, more recent data from a 32-week trial using long-acting exenatide in patients with MCI found no significant cognitive benefit [316].These mixed outcomes highlight the complexity of assessing the cognitive efficacy of GLP1 analogs, which may depend on patient population, treatment duration, baseline metabolic status, and the sensitivity of the cognitive endpoints used.

**Observational studies in AD and AD-like dementia patients**

When the risk to develop dementia is assessed, either no effects [347] or reduced odds ratio to develop dementia has been reported in diabetes patients on GLP1 analogs, showing that as the daily defined dose increases, the risk to develop dementia is reduced [320]. Also, GWAS performed on clinically diagnosed late-onset AD cases has reported a potential link between genetic variation in the gene that encodes GLP1 receptor and reduced risk to develop AD in these patients [317].

##### TZD

**Clinical trials in AD and AD-like dementia patients**

The assessment of TZD on cognition has also yielded mixed results. A large phase III study involving participants at risk of developing MCI due To AD found no significant differences over 5 years, and pioglitazone did not delay the onset of MCI [336]. Another phase III trial also supported the lack of significant effect of rosiglitazone (2 And 8 mg) in participants with mild to moderate AD, regardless of APOE genotype [330]. Similar outcomes have been reported with rosiglitazone over longer periods [304, 331, 332]. In contrast, a large study with rosiglitazone at 3 different doses over 24 weeks revealed significant cognitive improvement at the highest dose (8 mg) in APOE ε4 non-carriers, whereas APOE ε4 carriers experienced some cognitive decline [333]. In this line, a pilot study with mild to moderate AD or amnestic MCI patients treated for T2D with hypoglycemic medications or diet alongside pioglitazone showed cognitive improvements [302, 329, 348].

**Observational studies in AD and AD-like dementia patients**

Other set of studies have investigated the risk of developing dementia or AD among TZD users. It has been reported that TZD do not reduce the risk of developing AD in T2D patients, even up to 11 years after treatment initiation. Only minor, non-significant benefits have been observed when TZDs are combined with other antidiabetic medications [337, 340, 341]. In fact, long-term TZD use has even been associated with faster decline in immediate memory [381], and a cross-sectional Japanese study revealed an association between TZD use and the severity of dementia. However, the sample size was small and the study also reported an association between dementia severity and hypoglycemia, but not hyperglycemia [392]. In this line, a comparison between T2D patients who had ever used TZD and those who had never used them showed an increased hazard ratio and relative rate of dementia in TZD users [338]. Pioglitazone, in particular, has been associated with an increased risk of developing AD in T2D patients, with even worse outcomes observed in those receiving both pioglitazone and insulin. Importantly, the risk of developing AD increased with the cumulative defined daily doses of both pioglitazone and insulin too [342]. Despite this, a GWAS involving clinically diagnosed late-onset AD patients found that variations in *PPARG*, the gene encoding the TZD target protein, were associated with a trend toward reduced odds of developing AD [317]. Additionally, several studies have reported that TZD may reduce the risk to develop dementia in diabetes patients [134, 320, 347] And a lower incidence rate of new dementia diagnosis, per person-year, was observed in TZD users after a 5-year follow up [343]. In this line, pioglitazone may reduce the risk of dementia in a time- and dose-dependent manner in diabetic patients [339, 344]. Furthermore, a case report described cognitive improvement in a patient with mild memory complaints who Had been treated with a high dose of pioglitazone for 4 years, although this observational evidence should be interpreted with caution [334].

##### Biguanides

**Clinical trials in AD and AD-like dementia patients**

The number of clinical trials with biguanides is Limited. Previous studies Have shown that metformin administration for 16 weeks improves executive function in diabetic patients with MCI due to AD [305] And Another study revealed that, while some cognitive paradigms declined after 36 weeks, the group receiving metformin remained stable for some cognitive tests [382]. Also, a study with amnestic MCI overweight patients revealed that metformin over 12 months had no effect on a large battery of cognitive tests, although the total recall of the Buschke selective reminding test was improved [306]. The same study reported that metformin had a beneficial effect in younger participants, APOE ε4 carriers, those with lower HbA1c and those with higher insulin levels [306].

**Observational studies in AD and AD-like dementia patients**

Observational studies are more common, and improved cognition has been reported in long-term metformin users [351]. It seems that the time of initiation of metformin treatment, after diabetes diagnosis, is not associated with incident dementia compared to delayed or no initiation of pharmacotherapy [361]. Nevertheless, it has also been reported that long-term metformin users had a dose- and time- dependent higher risk of all-cause dementia, with a relevant implication of vitamin B12 [362]. Also, worse cognitive performances and increased risk of cognitive disfunction have been reported in older adults without dementia treated with metformin, even after adjusting for plasma B12 levels, both in longitudinal and cross-sectional studies [353, 363, 364]. Further assessment has also shown a higher risk to develop AD, as well as an association with the highest cumulative defined daily dose of metformin, even without a dose–response effect [135]. Similarly, long-term metformin users showed an increased risk to develop AD, although this effect could not be confirmed in the subgroup of metformin-only users, and there was no trend towards a higher risk as the number of prescriptions increased [337]. Additionally, metformin might be associated with faster rate of delayed memory decline in APOE ε4 carriers with AD, and with better immediate and delayed memory in APOE ε4 non‐carriers at the same time [381].

Alternatively, some studies have shown no association between biguanide, or metformin specifically, and the risk to develop dementia, other measures of cognitive aging or severity of dementia symptomatology [340, 347, 352, 357, 365, 382, 383, 392]. On the other hand, a large recent study with newly diagnosed T2D patients treated with metformin has revealed a reduction in the risk To develop dementia in Males, females as well as in patients under And over 70 years old [366]. In parallel, it has also been reported that metformin cessation might increase the incidence of dementia, independently of changes in HbA1 levels or use of insulin in a large study including race as covariable [367]. Given observed discrepancies, it Has been suggested that metformin initiation time might be determinant And a study comparing treatment delay revealed no association after 6.2–6.8 years follow-up. Interestingly, effects were similar in all race groups (white, black or Asian). Nevertheless, this study also acknowledges that patients initiating metformin monotherapy could Have started other drugs later or metformin after the first 6 months, making somehow hard to explain the actual effect of metformin treatment [368]. Contrarily, a cross-sectional study showed that metformin users with MCI due to AD have lesser cognitive decline [380] and similar observations have been reported in larger longitudinal and cross-sectional studies with AD and/or mixed dementia diabetes patients [349, 358]. Other studies have also provided compelling evidence regarding the neuroprotective role of metformin [320, 369–371], and these effects seem to be independent of APOE genotype [358]. While race/ethnicity has been used in some of these studies to normalize the baselines or as a covariable [371, 372] the specific effects on different race groups have not been addressed. Interestingly, when adjusted for the number of daily defined doses, the odds of developing dementia also show a gradual decrease [320], and metformin is associated with a lower risk to develop dementia in elderly diabetes patients in a dose–response manner [370]. In this line, when metformin is prescribed to T2D patients diagnosed ≥ 3 years before AD onset, over 10 years metformin exposure is associated with a reduced risk to develop AD, supporting the relevance of selecting appropriate time windows when evaluating risk factors for incident dementia [384].

##### SGLT2i, DPP4i and SU

**Observational studies in AD and AD-like dementia patients**

No clinical trials have addressed the effects of SGLT2i, DPP4i or SU in patients with AD o AD-like dementia. However, observational studies have explored these antidiabetic groups at different levels. The cognitive effects of SGLT2i have been scarcely studied. These antidiabetic drugs might be associated with a reduced risk of incident dementia [385], although another study reported no significant effects [347].

For DPP4i, the findings are also mixed. Some studies report no association with dementia risk [347], while others suggest potential cognitive benefits, such as a slower decline in delayed memory in AD patients [381]. Diabetes patients treated with vildagliptin (in combination with other antidiabetic drugs) for 26 weeks performed better in some cognitive domains [393]. Additionally, slower cognitive decline has been reported in diabetes patients with AD or mixed dementia who received DPP4i [349, 397]. Whereas vildagliptin alone does not affect the hazard ratio for developing dementia [394], other studies report that the odds to develop dementia are reduced in diabetes patients using DPP4i and that this reduction is associated with the number of daily defined doses [320]. Interestingly, other study has revealed that all individual DPP4i drugs analyzed (sitagliptin, linagliptin, saxagliptin, vildagliptin and alogliptin) have protective effects when taken over a year, with sitagliptin and saxagliptin showing benefits even with shorter treatment durations [395].

Long-term SU does not appear to be associated with a higher risk of developing AD in T2D patients who received between 1 And over 60 prescriptions (of 45–90 days) [337]. Similar outcomes have been reported in other large observational studies [320, 347, 357]. Likewise, SU use in control, MCI, AD or mixed dementia patients does not seem to significantly affect immediate delayed memory over time, as shown in both longitudinal and cross-sectional studies [349, 381]. In this Line, a cohort study revealed that after 2.5 years on SU treatment, Aβ42 levels could predict conversion to AD or MCI [352]. Moreover, SU monotherapy has been associated with a significantly reduced risk of developing AD in diabetes patients [340, 369]. A GWAS study further supports this, reporting that genetic variation in SU targets, KCNJ11 and ABCC8, which encode components of the ATP-sensitive potassium channel, are associated with a reduced risk of developing AD [317].

##### Insulin

**Clinical trials in AD and AD-like dementia patients**

Clinical trials involving insulin have used various formulations (short- and long-acting) as well as administration routes (peripheral and intranasal), which may help explain the variability in reported outcomes. When intravenously administered, insulin may have beneficial effects. A study found that insulin improved cognition in APOE ε4 homozygote AD participants [308], and an earlier study by the same group reported beneficial effects only in APOE ε4 non-carriers, with no effect observed in carriers [399]. Similarly, a small clinical trial involving memory-impaired patients, including those with AD, found that insulin administration facilitated delayed recall in APOE ε4 non-carriers compared with APOE ε4 carriers [400]. Besides, another small study in AD and amnestic MCI showed that insulin infusion led to a correlation between CSF norepinephrine levels and changes in paraphrase scores across when all participants are included (treated and control) [401]. Conversely, it has also been shown that intravenous insulin may worsen verbal episodic memory in both non-demented participants and cognitively impaired patients [409].

Intranasal insulin administration Has also been explored. In a study involving patients with AD or MCI, doses of 20 And 40 IU improved story recall in APOE ε4 non-carriers at both doses, whereas APOE ε4 carriers experienced significantly lower test scores at the 40 IU dose [402]. In this line, mild and moderate AD patients receiving a single dose of rapid-acting intranasal insulin (glulisine) made fewer errors on the trails B test. However, no significant effect was observed after APOE ε4 analysis, likely due to the small sample size [403]. A later clinical trial from the same group evaluating glulisine over 3 And 6 months in patients with AD or MCI reported no significant effects across a wide battery of cognitive tests, regardless of APOE ε4 status [404].

On the contrary, other studies have reported cognitive preservation or even improvement following intranasal treatment in MCI or AD patients when compared with participants receiving placebo [310, 313]. Additionally, higher CSF Aβ42 levels and tau/Aβ42 ratio are correlated with different cognitive tests after insulin treatment [310]. A later study from the same group further analyzed sex and APOE effect, though the outcomes are somehow hard To interpret. An overall improvement in story recall over time was seen both in men And women receiving 20 IU of insulin. However, only men improved at the highest dose under study (40 IU), and in other tests, only women showed benefit. APOE ε4 carriage did not predict treatment response at the cognitive or functional level. Still, APOE ε4-negative males benefited from the highest dose of insulin while APOE ε4-negative women had a worse response over time [405].

A larger trial examining 40 IU of intranasal insulin in AD patients found no significant effects [311]. Likewise, long-acting intranasal insulin (detemir) showed no overall benefit in a small study of AD patients. However, a significant treatment × time interaction was observed in APOE ε4 carriers, and verbal memory improved in insulin users. In contrast, APOE ε4-negative participants experienced a significant cognitive decline while on insulin [406]. Another small clinical trial combining vitamin D and intranasal insulin in AD patients reported initial improvements in cognitive tests, but these were followed by subsequent decline, indicating a limited and possibly transient benefit [407].

**Observational studies in AD and AD-like dementia patients**

The effects of long-term use on cognitive decline and risk of developing dementia Have been largely addressed, revealing An overall negative impact. The Rotterdam study already showed in 1996 that diabetes patients on insulin had an increased risk of developing dementia, specifically AD [417]. Similar outcomes have been reported afterwards examining insulin monotherapy or insulin in combination with other antidiabetic drugs [340, 342, 347, 357, 410, 412, 413, 418]. Insulin treatment has also been associated with greater dementia severity [392]. Supporting these clinical observations, a GWAS study analyzing genetic variation in the IR, found a trend toward increased AD risk in carriers of certain variants [317]. However, it is important to bear in mind that patients on insulin therapy generally have more severe diabetes than those not taking insulin, what may ultimately introduce some bias in these observations. Also, while some of these studies adjust for race/ethnicity as a covariate, the specific impact of insulin treatment across subgroups is often not analyzed in detail [413].

Even so, not all findings point to a detrimental effect. In a study on AD patients with diabetes mellitus, no significant differences were detected in the rate of cognitive decline between insulin users and non-users, patients with diet-controlled diabetes or those taking other antidiabetic drugs, including biguanides, SU, glinides or TZD [408]. Likewise, a larger cross-sectional study found no effects at cognitive level in diabetes patients suffering AD or mixed dementia [349], and other research reported that insulin use is not a significant predictor of conversion to AD or MCI [352]. An additional cross-sectional study, though limited in detail, has also supported this lack of effect [414]. In a cohort of patients with new-onset diabetes who Had suffered hypoglycemia And were treated with oral hypoglycemic agents, with or without insulin, the use of insulin for more than 180 days was not associated with increased dementia risk [350]. On the other hand, a few studies have reported protective effects of insulin. In this sense, short-term insulin use (fewer than 60 prescriptions of 45–90 days each) was associated with a reduced risk of developing AD [337]. Strikingly, a large nationwide study with diabetes patients showed that insulin users had lower odds of developing dementia, and that this protective action was not associated with the number of daily defined doses [320].

##### Other antidiabetic drugs, combined therapies and treatment comparisons

**Clinical trials in AD and AD-like dementia patients**

Only a limited number of clinical trials have directly compared the effects of different antidiabetic treatments yielding mixed results. In this sense, it has been reported that cognitive decline might be worsened in patients who only receive oral antidiabetic drugs when compared with those on combined therapy (oral antidiabetic + insulin) [419]. In another study, a significant increase in short-term memory and composite memory z-score has been reported in prediabetes patients on metformin who also received liraglutide, although differences were compromised after adjusting for baseline values [321]. Also, large studies have reported a reduced risk of overall dementia, AD, vascular dementia and MCI when metformin is compared with SU [300, 301], while metformin + rosiglitazone treatment stabilized cognitive scores over a 36-month period [382]. Given the range of therapies not included as first-line approaches, as well as the use of combination therapies, a summary of the Outcomes is provided in Table 4.

**Observational studies in AD and AD-like dementia patients**

Given the complexity of diabetes management, many studies have analyzed the effects of combined hypoglycemic therapies, often comparing different antidiabetic treatments without a placebo, untreated, or true control group. On top of this, in some studies oral hypoglycemic agents are considered a single group without distinguishing individual treatments. It has been reported that both oral hypoglycemic drugs and insulin do not significantly interfere with the risk to develop dementia when included as covariables in a longitudinal observational study [415]. Nevertheless, early findings, such as those from the Rotterdam Study, indicated that diabetes patients on oral hypoglycemic agents had an increased risk of developing dementia and AD specifically [417] and subsequent longitudinal studies have confirmed these associations [410, 418, 420].

GLP1 analog semaglutide administration to T2D patients for a year was associated with reduced cognitive impairment compared to sitagliptin, and both cognitive decline and dementia incidence were lower than in patients treated with glipizide. No significant differences were observed when semaglutide was compared with empagliflozin [322]. Also, a large recent study has shown that semaglutide is associated with significantly reduced risk of first-time AD diagnosis in T2D patients when compared with those prescribed other antidiabetic medications, including insulin and other GLP1 analogs. Notably, the protective effect of semaglutide also appears stronger in women [323]. Similarly, long-term follow up to T2D patients has revealed that GLP1 analogs are associated with a lower risk of dementia when compared with SU and DPP4i in older individuals [324].

Studies evaluating combined therapies have also revealed mixed results. Certain combinations, such as a DPP4i + SU or DPP4i + insulin were significantly associated with dementia severity, while others, such as α-glucosidase inhibitors, TZD + insulin, or TZD + insulin + SU, showed no association when adequate metabolic control was achieved (HbA1c < 7%) [392]. Alternatively, other large study with less common antidiabetic agents has reported no significant effect on AD risk from α-glucosidase blockers, non-SU insulin secretagogue monotherapy or combination therapies [340].

Given the extended use, metformin has often been the reference drug in comparative studies, yet the results remain inconclusive. SU and TZD users may have worse prognosis to develop dementia than metformin users [338, 373–375]. However, race-specific sub analyses show that metformin use is associated with lower dementia hazard ratios in Caucasian populations, but not in African American or Asian patients [375]. While the number of participants was lower, this may explain some inconsistencies observed in the literature. Also, patients taking metformin perform better than those on SU, although differences might depend on age and race [376].

Similar protective outcomes have been reported in comparisons with other glucose-lowering agents [325, 377] and the dementia risk reduction appears dose-dependent [378]. Race also plays a role: in one study, metformin significantly reduced dementia risk in African American patients aged 50–64, while lower dementia rates were observed in both African American And white patients aged 65 to 74, supporting the relevance of the race and age in these studies [379].

Some smaller retrospective studies have found that metformin combined with vildagliptin led to better cognitive performance than metformin alone in elderly patients with MCI [391]. Similarly, and significantly higher better cognitive scores have been reported for both insulin and sitagliptin users, when compared with metformin use alone [396]. Also, combination therapy with metformin + SU was associated with a reduced dementia risk compared to either agent alone a study with T2D [369]. A comparative study involving metformin, DPP4i, GLP1 analogs, SU, and insulin found that all combinations, except those including SU, were associated with lower odds ratios for developing dementia [320]. Alternatively, SU agents such as glyburide, nateglinide and glipizide treatments may result in higher adjusted odd ratios to suffer dementia, whereas lower risks are observed for rosiglitazone, exenatide, liraglutide, dulaglutide and sitagliptin when compared with metformin [326].

Overall, most of studies indicate that metformin is associated with reduced cognitive decline compared to other antidiabetic agents. Cognitive decline is reduced in metformin users when compared with non-users, whereas insulin and SU users may have larger point-wise decrements when compared with DPP4i users [349]. Likewise, diabetes patients taking metformin perform better than those on other oral hypoglycemic drugs, as reported both in cross-sectional and longitudinal studies [354, 411] or on metformin + insulin [411]. In this line, a lower risk of dementia has been detected in individuals aged ≥ 65 treated with pioglitazone when compared with other second-line options, in metformin-based dual therapy. Further assessment also revealed that the metformin + pioglitazone combination showed lower dementia risk than metformin + rosiglitazone, and other combinations (with SU, DPP-4i, insulin, meglitinide, or acarbose) were also beneficial [422].

Nonetheless, conflicting results persist. Some studies report that SU and SU combinations (metformin + SU or SU + TZD) increase the risk of all-cause and vascular dementia, while TZD monotherapy and metformin + TZD combination appear protective. With prolonged use, no differences were observed with SU or combined metformin + TZD therapy, while TZD became more protective [335], making the results hard to interpret. On the other hand, it has also been reported that while SU monotherapy may increase risk to develop dementia, in combination with other antidiabetic drugs the risk of dementia is reduced [421]. Other comparisons have revealed that TZD may lower dementia risk compared to α-glucosidase inhibitors [345].

Direct comparisons between SGLT2i and DPP4i show lower dementia risk for the SGLT2i group [386–388]. Interestingly, when SGLT2i were individually analyzed, dapagliflozin had the lowest dementia risk, followed by empagliflozin, whereas no association was detected when canagliflozin was compared with DPP4i [387]. When analyzed together (dapagliflozin and empagliflozin), they have a beneficial effect on the cumulative incidence of dementia, and AD specifically, compared with GLP1 analog dulaglutide [327]. SGLT2i also reduce the risk of developing dementia compared with SU use in T2D patients, with effects varying by race/ethnicity and chronic kidney disease status [389]. In a study analyzing patients on SGLT2i monotherapy or in combination with other antidiabetics (including metformin, SU, meglitinide, DPP-4i, TZD, α-glucosidase inhibitors, or insulin), SGLT2i use was associated with reduced risk of both AD and all-cause dementia when compared with participants on oral antidiabetic drugs other than SGLT2i, with or without insulin, for over 90 days [390]. Likewise, SGLT2i + metformin reduces the risk of dementia when compared with patients on DPP4i + metformin or those on GLP1 analogs + metformin [328].

Finally, data from large-scale studies suggest that biguanide monotherapy or its combination with other antidiabetics reduces dementia risk. DPP4i appear protective both as monotherapy and in combination. When meglitinide or TZD are addressed in combination with other antidiabetic drugs, the risk of dementia seems to be reduced. Also, when patients receive metformin + TZD, metformin + SU + meglitinide, metformin + SU + α-glucosidase inhibitor, metformin + SU + TZD, metformin + meglitinide + TZD or metformin + α-glucosidase inhibitor + TZD higher risks of dementia are detected, while combinations including DPP4i reduced it, as in metformin + SU + DPP4i or metformin + α-glucosidase inhibitor + DPP4i in comparison with metformin alone [421]. Despite the volume of data, the diverse drug effects and complex interactions make the interpretation of outcomes challenging (Table 4).

### Clinical studies closing remarks

While there is extensive bibliography analyzing the effects of antidiabetic drugs in patients and the risk to develop dementia, the complexity of the outcomes makes it difficult to decipher the actual take-home messages in many cases. Therefore, the transition from preclinical success to clinical application requires further investigation.

### Limitations of the study

One limitation of this review is the absence of a formal risk of bias assessment. This was due to the inherent difficulties in evaluating the methodological quality of preclinical studies, particularly those involving animal models, which often do not provide enough detailed reporting for a comprehensive bias evaluation. While we included all studies that met our eligibility criteria, we acknowledge that this approach may limit our ability to fully evaluate the methodological rigor of the included studies. Despite this, the inclusion of a broad range of studies ensures a more inclusive overview of the evidence, spanning both preclinical and clinical studies. We have explicitly acknowledged this limitation in the manuscript, encouraging readers to interpret the findings with caution. The findings highlight key trends in the efficacy of antidiabetic drugs for AD and AD-related dementia, particularly the promising role of GLP1 agonists or metformin, and suggest potential avenues for repurposing these drugs as treatments for AD.

On the other hand, the issue of brain delivery potential remains largely unexplored in studies of antidiabetic drugs in AD, leaving uncertainty about how these treatments exert their effects. While some of these drugs may have limited direct access to the brain, their neuroprotective effects could result from direct actions within the CNS, indirect systemic effects, such as modulation of systemic inflammation or improvement of metabolic health, or a combination of both. Clarifying whether these drugs act directly on the brain or through systemic effects influencing neurodegeneration is crucial to optimizing therapies, and therefore further research is needed to determine the contribution of each mechanism.

Finally, as stated above, in most clinical studies, the number of patients tends to be low, whereas in studies assessing the risk to develop dementia, significant cofactors such as sex, race, ethnicity, or *APOE* genotype or residual confounding are obviated in many cases significantly limiting the significance of the observations. Likewise, AD specific therapies (acetylcholinesterase inhibitors or memantine) or other pharmacological treatments are not always detailed or included as cofactors in the analysis. Also, specific cognitive tests and criteria are largely variable, as the treatments, combined therapies, doses or length of the follow-up studies. In this sense, combined therapies are usually introduced as metabolic control becomes harder to achieve what ultimately results in a study including patients with more severe complications. In this line, in many cases it is difficult to discern the influence of observational bias, preexisting conditions, or the severity of diabetes in the populations under study. Also, most studies do not report the raw data required for meta-analysis, limiting the calculation of standardized effect sizes and preventing robust quantitative synthesis. In other cases, antidiabetic drugs are sometimes grouped together without individual analysis or comparison to specific AD therapies, while, in others, treatments are compared with each other lacking a control group, making it hard to determine the actual meaning of the results.

## Supplementary Information

Supplementary Material 1: Supplementary Table 1. Complete reference library.

Supplementary Material 2: Supplementary Table 2. Preclinical studies.

Supplementary Material 3: Supplementary Table 3. Clinical studies.

## Data Availability

No datasets were generated or analysed during the current study.

## Abbreviations

4-HNE: 4-Hydroxynonenal
8-OHdG: 8-Hydroxy-2'-deoxyguanosine
ACh: Acetylcholine
AChE: Acetylcholinesterase
AD: Alzheimer's disease
AGEs: Advanced glycation end products
AMPK: 5’ Adenosine monophosphate-activated protein kinase
ApoE: Apolipoprotein E
APP: Amyloid precursor protein
Aß: Amyloid ß
BACE1: ß-secretase 1
BBB: Blood–brain barrier
BDNF: Brain-derived neurotrophic factor
ChAT: Choline acetyltransferase
COX-2: Cyclooxigenase-2
CREB: CAMP-response element binding protein
CSF: Cerebrospinal fluid
DPP4i: Dipeptidyl peptidase IV inhibitors
ERK: Extracellular-signal regulated kinase
GFAP: Glial fibrillary acidic protein
GIP: Gastric inhibitory polypeptide
GLP1: Glucagon-like peptide-1
GSK3ß: Glycogen synthase kinase 3ß
GWAS: Genome-wide association study
HFD: High-fat diet
Iba1: Ionized calcium-binding adapter molecule 1
IDE: Insulin-degrading enzyme
IFN-γ: Interferon gamma
IL: Interleukin
iNOS: Inducible nitric oxide synthase
IR: Insulin receptor
IRS: Insulin receptor substrate
IκBα: NF-kappa-B inhibitor alpha
LDH: Lactate dehydrogenase
LRP1: Low density lipoprotein receptor-related protein 1
LTD: Long-term depression
LTP: Long-term potentiation
MAPK: Mitogen-activated protein kinase
MAP2: Microtubule-associated protein 2
MCI: Mild cognitive impairment
MDA: Malondialdehyde
MRI: Magnetic resonance imaging
mTOR: Mammalian target of rapamycin
MWM: Morris water maze
NeuN: Neuronal nuclei
NO: Nitric oxide
NOR: Novel object recognition
PET: Positron emission tomography
PICO: Population, Intervention, Comparison and Outcome
PI3K: Phosphatidylinositol 3-kinase
PPAR-γ: Peroxisome proliferator activated gamma
PreDM: Prediabetes
PRISMA: Preferred Reporting Items for Systematic Review and Meta-Analyses
PSD95: Post-synaptic density protein 95
RAGE: Receptor for advanced glycation end products
ROS: Reactive oxygen species
SGLT2i: Sodium-glucose cotransporter 2 inhibitors
SOD: Superoxide dismutase
STZ: Streptozotocin
SU: Sulphonylureas
T1D: Type 1 diabetes
T2D: Type 2 diabetes
TBARS: Thiobarbituric acid reactive substances
TNF-α: Tumor necrosis factor alpha
TZD: Thiazolidinediones
